# Evaluation of 16S rDNA-Based Community Profiling for Human Microbiome Research

**DOI:** 10.1371/journal.pone.0039315

**Published:** 2012-06-13

**Authors:** Jumpstart Consortium Human Microbiome Project Data Generation Working Group

**Affiliations:** Institute for Genome Sciences, University of Maryland School of Medicine, United States of America

## Abstract

The Human Microbiome Project will establish a reference data set for analysis of the microbiome of healthy adults by surveying multiple body sites from 300 people and generating data from over 12,000 samples. To characterize these samples, the participating sequencing centers evaluated and adopted 16S rDNA community profiling protocols for ABI 3730 and 454 FLX Titanium sequencing. In the course of establishing protocols, we examined the performance and error characteristics of each technology, and the relationship of sequence error to the utility of 16S rDNA regions for classification- and OTU-based analysis of community structure. The data production protocols used for this work are those used by the participating centers to produce 16S rDNA sequence for the Human Microbiome Project. Thus, these results can be informative for interpreting the large body of clinical 16S rDNA data produced for this project.

## Introduction

The human body is host to an abundant and complex diversity of microbial life [1]–[7]. With estimates of the total species that inhabit an individual ranging well into the thousands [8], it is evident that we understand only a small component of the human microbiome. There is growing recognition, however, that resident microbial communities influence human nutrition, development and disease [9]. For example, dysbiosis of the microbiome has been implicated in many phenomena, including obesity, inflammatory bowel diseases [10], [11], dermatitis and atopic diseases [12], [13], bacterial vaginosis and pre-term birth [14], [15].

The NIH Roadmap Human Microbiome Project (HMP) has undertaken a large-scale, culture-independent census of the microbiota of healthy adults that will describe the members of human-associated communities and establish the extent to which these communities, or their constituents, are shared between individuals and body sites [16]. The HMP is publicly releasing sequence from approximately 12,000 samples that survey 15 (male) and 18 (female) body sites from 300 healthy adults [17].

For the HMP, the prolonged period over which the samples were collected and sequenced, and the participation of multiple sequencing centers, created an unprecedented need for standardization and benchmarking of 16S rDNA (16S) profiling methods. In the course of preparing for the data production phase of the project, the sequencing centers generated abundant sequence data from a synthetic microbial community, as well as from a set of clinical samples from several body regions. Data generated from the MC were invaluable for the development of ChimeraSlayer, a tool for detecting chimeric 16S rRNA reads [18] and are intended to be a useful resource for continued development and assessment of analysis tools. Here, these data enabled standardization and cross-validation of the data production methods used by the HMP consortium and, more significantly, enabled investigation into the performance characteristics of the sequencing technologies used and the influence of sequence errors on the interpretation of 16S rDNA community profiling.

## Results

**Figure 1:**
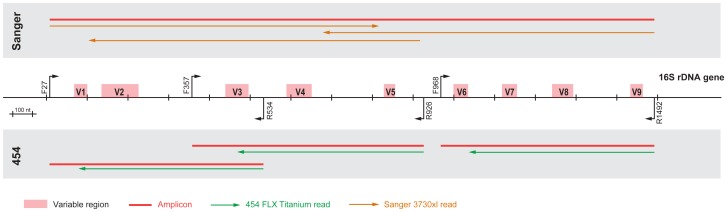
Overview amplicons and reads generated for both the 3730 and 454 sequencing. On a schematic representation of the 16S rDNA gene, the known variable regions and the primers used in this study are indicated. Positions and numbering are based on the *Escherichia coli* reference sequence. The amplicons generated by each primer set are marked in red, and sequencing directions and expected lengths are indicated in orange for 3730 and green for 454.

When the HMP project was initiated, ABI 3730 and 454 FLX Titanium platforms were both in use at the participating centers. Thus, the analysis herein frequently compares both data types. In the process of establishing molecular and analytic workflows, the centers constructed a synthetic, or ‘mock’ community (MC) composed of 21 archaeal/bacterial species representing 18 genera (Materials and Methods). All MC members have finished reference genomes and represent a range of %GC content and phylogenetic diversity. This MC provided a defined standard to benchmark the accuracy of our data with respect to community composition. In addition, comparison of our 16S data to the reference sequences allowed us to directly assess sequence quality. All centers sequenced the MC in duplicate (3730) or in triplicate (454). Multiple amplicons were targeted for sequencing, spanning different regions of the 16S rDNA (Fig. 1). The protocols used to produce data and the number of reads represented in the results below are provided in the supporting information (Tables S1, S2, S3, S4, S5 and Protocol S1 and Protocol S2).

### Community Composition: BLAST Versus Naïve Bayesian Classification

**Figure 2:**
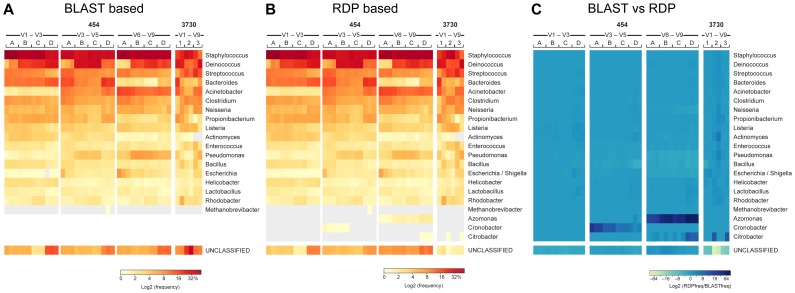
Minor differences in classifiability as measured by the RDP Classifier and a BLASTn-based approach . The left panel shows classification based on BLASTn against reference sequences of the MC members. A sequence is classified if it has >95% global sequence identity with one of the reference sequences and >90% of read is contained in the alignable region. Results are shown as a heatmap depicting the frequency values, using a binary logarithm scale. The middle heatmap illustrates frequency values of taxa identified using the RDP classification tool, applying an 80% confidence cutoff. Right panel shows the difference between RDP and BLASTn based classification, with a heatmap representing the ratio of observed genus-level frequency data (RDP) over expected genus-level frequency (BLASTn) for each of the MC members using a binary logarithm scale.

**Figure 3:**
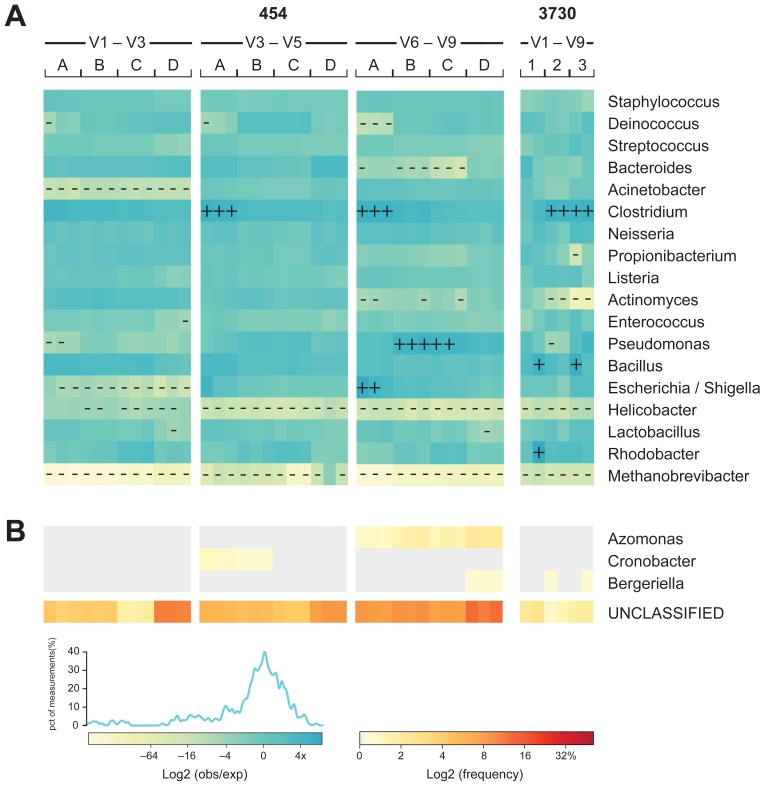
Mock community-based accuracy of community representation compared across technology and 16S window. The MC was sequenced by different centers on both 3730 and 454 platforms. Each sequencing trial is represented as a column. For 3730 sequencing of the V1–V9 window, amplicons derived from a common amplification protocol were sequenced with short capillaries (1), long capillaries (2), and three reads per clone (3). 454 sequencing was performed by four centers (A, B, C, and D) with three 16S windows (V1–V3, V3–V5, and V6–V9). (A) The observed genus-level frequency data over expected genus-level frequency ratio for each of the MC members is shown as a heatmap using a binary logarithm scale. The expected frequency ratio is based on the whole genome coverages inferred from mapped Illumina WGS reads to the MC reference genome sequences. Genera with observed frequencies differing more than four-fold from expected are marked with + or – for over- or under-representation, respectively. (B) The fraction of misclassified (0.1% of the total combined data set) and unclassified (4.6% of the total combined data set) sequences displayed as a frequency heatmap. The frequency values are depicted as a binary logarithm scale.

We first examined the observed relative abundance of each community member by using BLAST to compare all reads against a reference set of 16S sequences that was derived from deeply sequenced 16S clone libraries prepared from each organism in the MC and which captured the sequence diversity at all 16S loci. We were able to reliably detect all MC members in all data sets except for the sole archaeal member, *Methanobrevibacter* (Fig. 2A); this was anticipated, since primers that target bacterial 16S sequences diverge from the sequences found within Domain Achaea (Fig. 3D).

We reexamined the MC data using a naive Bayesian classification-based regime appropriate for samples of unknown community composition (Materials and Methods). For this analysis, reads that were greater than 200 nucleotides in length and classified with 80% confidence to a genus using the RDP classifier [19] were assigned a genus-level taxonomic identifier and binned based on this assignment (Fig. 2B). The relative community composition reported by the classification analysis agreed well with the BLAST-based analysis (Fig. 2C), However, in our BLAST- and classification-based analyses of the MC data, 4.2% of the total reads generated across many samples did not match any input MC 16S (Fig. 2A and 2B). Also, for some data sets, there was a subset of reads that classified to organisms that were not part of the MC (Fig. 2B and 3B). This observation is considered in greater detail below.

### Community Composition: Quantitative Accuracy

We then sought to establish the quantitative accuracy of the observed community compositions across each technology. While preparing this analysis, we observed concerning disparities between the calculated expected abundances of the members of the MC previously described [18] and the results we were obtaining. We could not confidently establish that the calculated expected abundances were correct. We elected to be circumspect and obtain an independent estimation of the MC composition. To do so, total DNA from the MC was sequenced using a whole genome shotgun (WGS) approach. WGS methods mechanically shear genomic fragments for sequencing and, therefore, would not be subject to sequence-targeted amplification biases. The abundance of each organism was determined from a count of WGS sequence reads mapped to each reference genome (Materials and Methods). The relative abundance, as determined by WGS was used here as the reference MC composition.

Data from all MC members, across all experiments, both 3730 and 454, exhibited 1.4-fold difference from expected and only 1.07-fold (Figure 3) when results from *Methanobrevibacter* were excluded from the analysis. We also observed that 454-generated data were slightly more accurate than data generated using 3730 (1.035-fold versus 1.27-fold) and that 454 data generated using the V3–V5 primers were statistically significantly more accurate relative to data generated using the other two primer pairs (0.8-fold for V3–V5 compared to 1.14-fold for both V1–V3 and V6–V9; p<0.01 in all cases).

**Figure 4:**
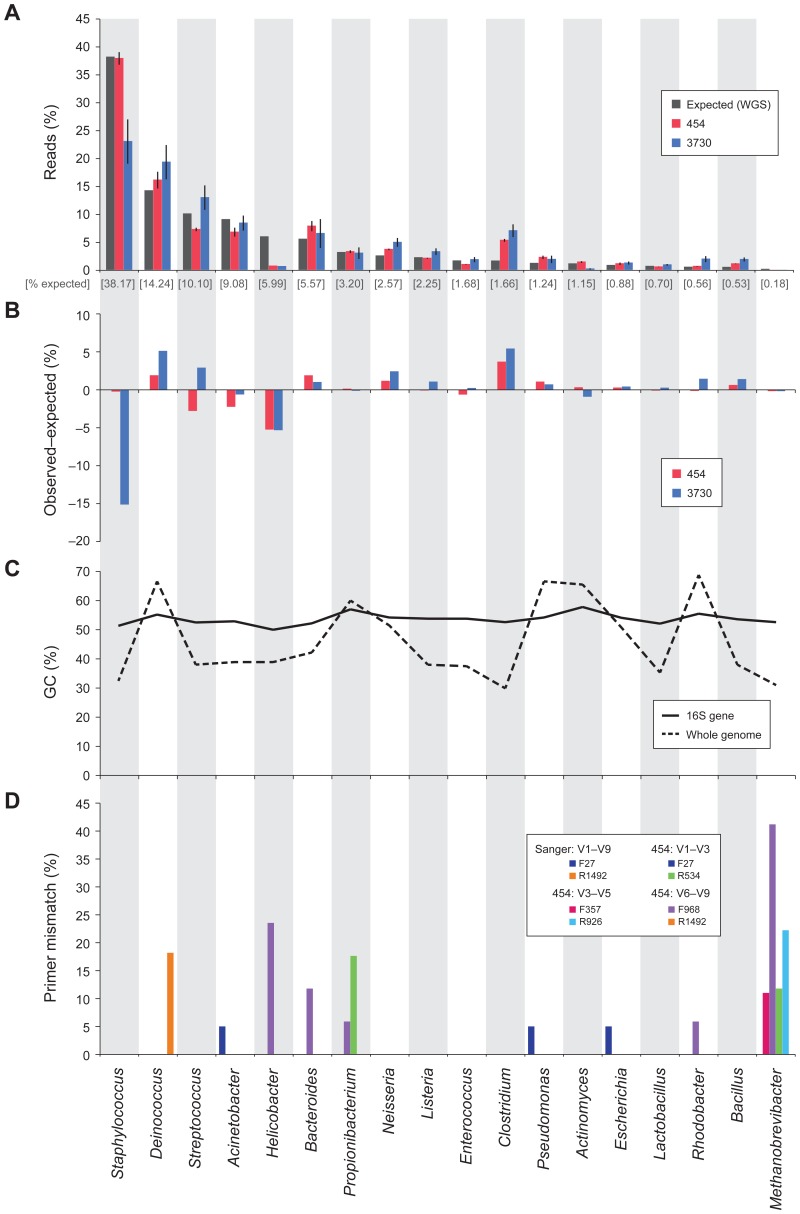
Deviation from expected in the 16S based Mock Community member representation can partially be explained by primer mismatch, not by %GC differences. The 20 bacterial organisms of the Mock Community are represented by corresponding genus (n = 18) along the bottom of the figure, and across the four panels (DNA from *Candida albicans* was included in this mock community, but not shown here). (A) The distribution of reads over the 18 genera; The expected frequencies (grey) in the community determined by whole genome shotgun (WGS) sequencing and classified by mapping to reference genomes using BWA, and the observed frequencies determined by 454 reads (red) or 3730 sequences (blue), classified by BLASTn. Error bars indicate standard error from technical replicates. (B) Deviations from expected frequencies as calculated by subtracting expected % from the observed. (C) The average %GC is shown for all its 16S genes, and for their whole genomes. (d) The lowest percent mismatch between primer used in production protocols (Protocols S1 and S2) and any 16S gene copy is shown for each organism; primers are grouped by sequencing technology and 16S window.

While overall accuracy was generally very good, there were notable differences from expected compositions among members of the MC. There were differences in MC representation that correlated with sequencing center or with window (Fig. 3A). For example, *Acinetobacter* was consistently underrepresented by window V1–V3 across all centers, while *Deinococcus* was underrepresented by only one center. We were unable to identify factors accounting for differences between centers. *Staphylococcus* was represented accurately among all experiments, regardless of window, platform or center [0.23-fold]. At the other extreme, the abundance of *Helicobacter* was greatly underrepresented across all experiments [3.48-fold]. In an attempt to explain the variation, we explored %GC content variation among the MC members and found no obvious correlation between %GC content of either the 16S or whole-genome sequences that could explain differences in the accuracy of MC member estimation (Fig. 4C). However, we did observe that the amplification primers used contained mismatches to the 16S gene sequence for several of the genera where lower than expected frequencies were observed (Fig. 4D). For example, nearly 15% of bases in the V6–V9 forward primer contained mismatches to *Bacteroides* and nearly 25% to *Helicobacter*. Percentage of primer mismatch bases did not, however, correlate with all inaccuracies; some genera (e.g., *Clostridium*) were consistently overrepresented.

### Analysis of Unclassified and Misclassified Reads

**Figure 5:**
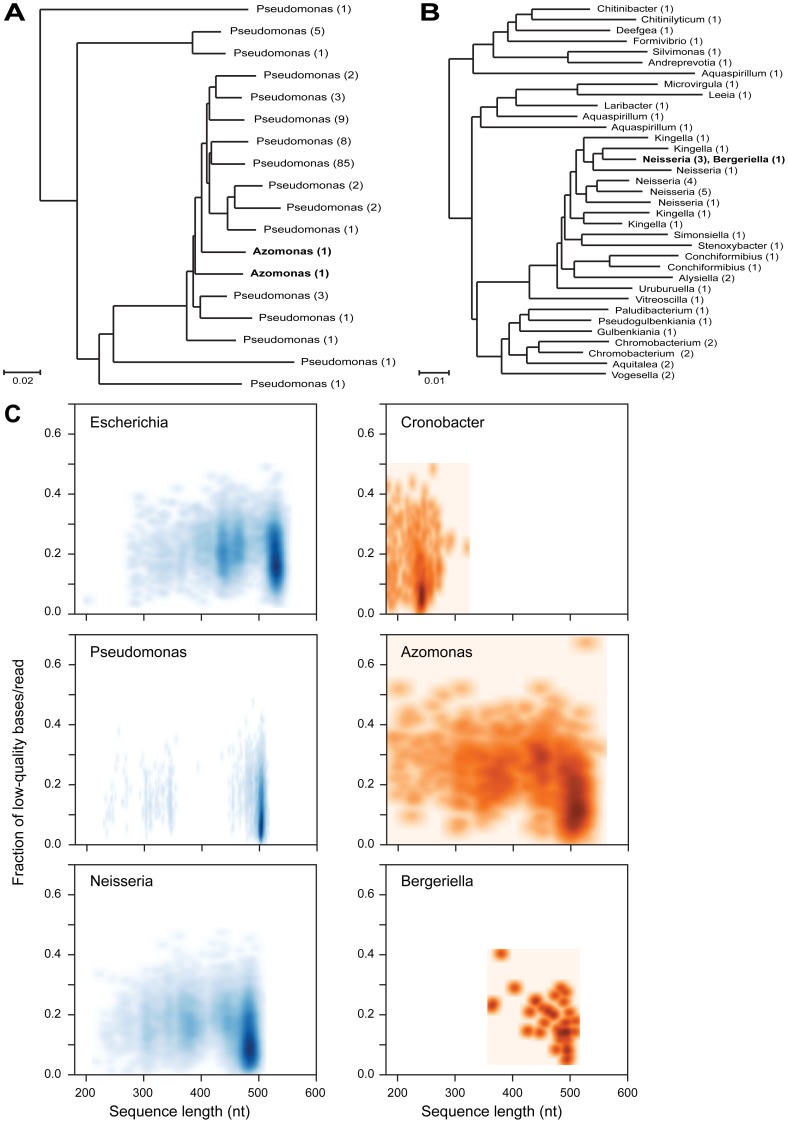
Illustration of how flawed taxonomic schemes and sequence quality can result in incorrect classifications. The phylogenetic trees were created starting from the full-length reference sequences that were used to train RDP’s taxonomic scheme version 5 for *Pseudomonas* and *Azomonas* (A), and *Neisseriaceae* (B), respectively. These sequences were clustered into 3% OTUs with mothur and representatives for each OTU were selected for building a tree with FastTree. The number of sequences belonging to each OTU is indicated in brackets.(C) Scatter density plots of percent low quality (QV<20) bases per read versus read length is shown for the misclassified reads (red) compared to their correctly classified counterparts (blue).

Further exploration of reads classified as *Azomonas*, *Cronobacter* and *Bergeriella* (Figs. 2B and 3B) revealed that poor read quality, as well as inconsistencies within the underlying taxonomic scheme used in the applied classification regime, resulted in misclassification of reads from MC members (Fig. 5). Close phylogenetic relationships between differently named sequences within the taxonomic scheme we utilized resulted in misclassification of *Pseudomonas* and *Neisseria* as *Azomonas* and *Bergeriella*, respectively (Figs. 5A and 5B), while prematurely truncated *Escherichia* reads were misclassified as *Cronobacter* (Fig. 5C). We expect phylogenetic artifacts to diminish as taxonomic schemes continually improve. We also note that others have recently compared the impact of selecting alternate taxonomic training sets on classifiability and abundance estimation [20].

Lack of accurate PCR amplification explained the majority of the unclassified 16S reads. Approximately 80% of the unclassified 3730 and 454 data (Figs. 2A, 2B and 3B) were flagged as chimeric sequences. We then compared non-chimeric, unclassified reads to their parent reference sequences and determined the frequency of substitutions, insertions and deletions within each read. The error rate within unclassified MC reads was significantly higher than that of classified reads. For both platforms, the error rate for unclassified reads was up to 10-fold higher for 3730, and 2-fold higher for 454 than for the classified reads (data not shown). These errors could arise during PCR amplification or during sequencing.

**Figure 6:**
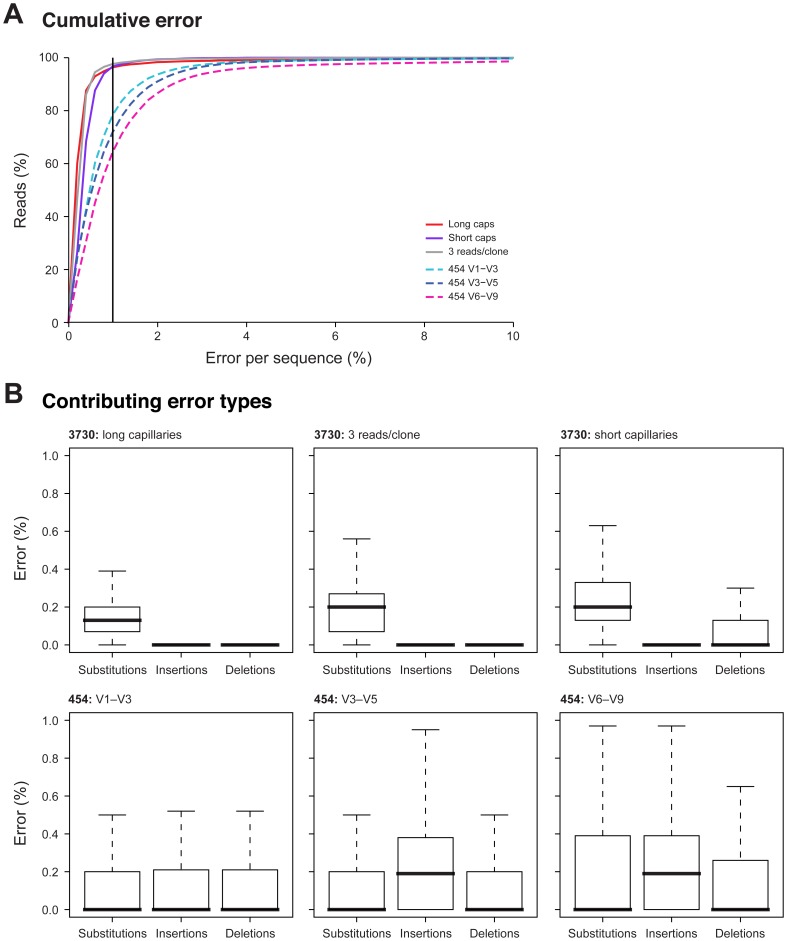
454 sequences have a higher error rate, mainly resulting from an increased insertion and deletion rate. (A) For all the quality and chimera filtered 3730 and 454 sequences generated for the MC sample, an alignment-based estimation of errors, including insertions, deletions, and substitutions was performed. For each of the different sequencing approaches, the cumulative frequency distribution of the percent error per sequence is shown for assembled 3730 sequences generated with short capillaries (green), long capillaries (red), and three reads per clone (yellow), and 454 reads spanning the variable regions V1–V3 (light blue), V3–V5 (dark blue), and V6–V9 (fuchsia). A vertical line at 1% was added as a visual aid for upper limit of an acceptable error threshold. (B) Boxplots show the average percentage of errors per read, per sequence approach and per error type, including substitutions, insertions, and deletions. Outliers are not shown.

**Figure 7:**
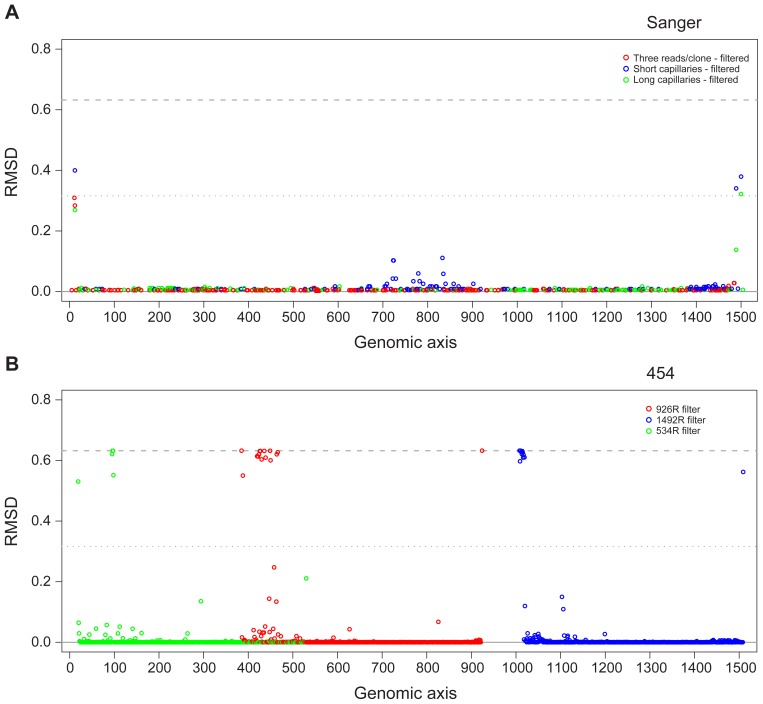
Error by position profiles indicate hotspots for error. To visualize where sequencing errors were concentrated along the length of the 16S sequence for each sequencing technology, a root mean square deviation (RMSD) plot was generated for (A) 3730 sequence and (B) 454 read data. The RMSD plot is a graphical representation of the differences in nucleotide distribution between a reference sequence and the samples of interest, for each position along the length of the reference. This figure shows the results for *Neisseria meningitidis* specifically, but is representative of the profiles observed for the other strains in the MC.

We then examined the cumulative error frequency distribution and frequency of error types for the six read types we produced (Figs. 6A and 6B). We also examined error by position within the reads (Fig. 7). Each platform presented a distinct error type profile. 3730 sequencing tended to generate substitution errors at an approximate average frequency of 0.2% errors per read; 454 sequencing, on the other hand, generated insertion errors at a similar frequency. These differences suggested that most sequence error was generated during sequencing rather than during PCR amplification. We also observed that sequences derived from the V1–V3 window contained fewer insertion errors than those from either V3–V5 or V6–V9 windows. However, we do not claim that this is a generalizable result. Homopolymeric bases are a known source of insertion errors in pyrosequencing [21]. The unique composition of the MC likely accounted for this observation. For example, the two *Staphylococcus* species comprised approximately 38% of the expected MC composition and contain homopolymeric 6-mers in the V3–V5 window, but not in either the V1–V3 or V6–V9 windows (data not shown). The position of homopolymers within the read may also contribute to the observed insertion frequencies. Errors tend to occur at the tail end of 454 reads and for sequences derived from paired 3730 reads, errors tend to occur in the first few bases of the parent reads. Where 3730 sequences were derived from short capillary reads errors are observed where the overlapping regions are assembled.

Unfortunately, error rates cannot be used to pre-filter inaccurate reads unless the parent reference sequences are known. We attempted to determine simple read quality characteristics that could be used to identify inaccurate sequences without relying on more advanced read filtering or denoising approaches. We point to other groups actively advancing methods for data filtering [22], [23] and to a comparison of approaches using the mock community [24]. We focused on two metrics: read length and the percent of low-quality bases per read. For a fraction of the non-chimeric, unclassified data there was a general relationship between sequence quality, length and error rate; erroneous reads tended to be shorter and had more low-quality bases (data not shown). These two measures of sequence quality alone, however, were not predictive of true error rate for 60% of 3730 and 40% of 454 reads that were not classified. Thus, simple filtering metrics that would remove all erroneous and unclassifiable sequences from both 3730 and 454 16S data were not apparent.

### Classifiability

We explored, first, how different data types, differentiated by technology or 16S window, impacted our ability to classify data for the MC and, second, how this compared to clinical samples taken from four body regions: gut, oral cavity, skin and vagina. In the process of removing detectable chimeras from all data sets prior to taxonomic analysis, we observed that the proportion of chimeras varied markedly between different samples and sequencing platforms (Table 1). It is known that community composition as well as amplification template concentration can influence the frequency of chimera formation in 16S amplification reactions [18]. Among the body sites tested, the stool and oral communities had the highest proportion of chimeras overall, while 3730 sequences contained notably fewer chimeras than did the 454 data (Table 1). There was only a modest difference in chimera rates detected across the different 16S windows sequenced by 454, with V1–V3 having the greatest and V6–V9 the lowest chimera content.

**Table 1:** Chimera rates in 16S data sets.

Samples	% Observed Chimera content			
	ABI3730	454 FLX Titanium		
	V1–V9	V1–V3	V3–V5	V6–V9
MC	5.99±3.07	14.26±10.34	14.75±9.45	13.49±8.52
gut	7.71±6.46	22.90±8.56	16.03±2.86	17.76±3.76
oral	7.22±6.35	20.55±11.73	10.98±4.01	9.10±5.02
skin	3.49±5.77	11.15±1.36	7.51±2.49	5.73±1.69
vaginal	6.31±6.64	12.60±6.70	6.62±3.51	3.00±1.65

* Values are averages ± STDEV calculated from multiple replicates of MC, and from replicates of multiple clinical samples originating from different body sites.

**Figure 8:**
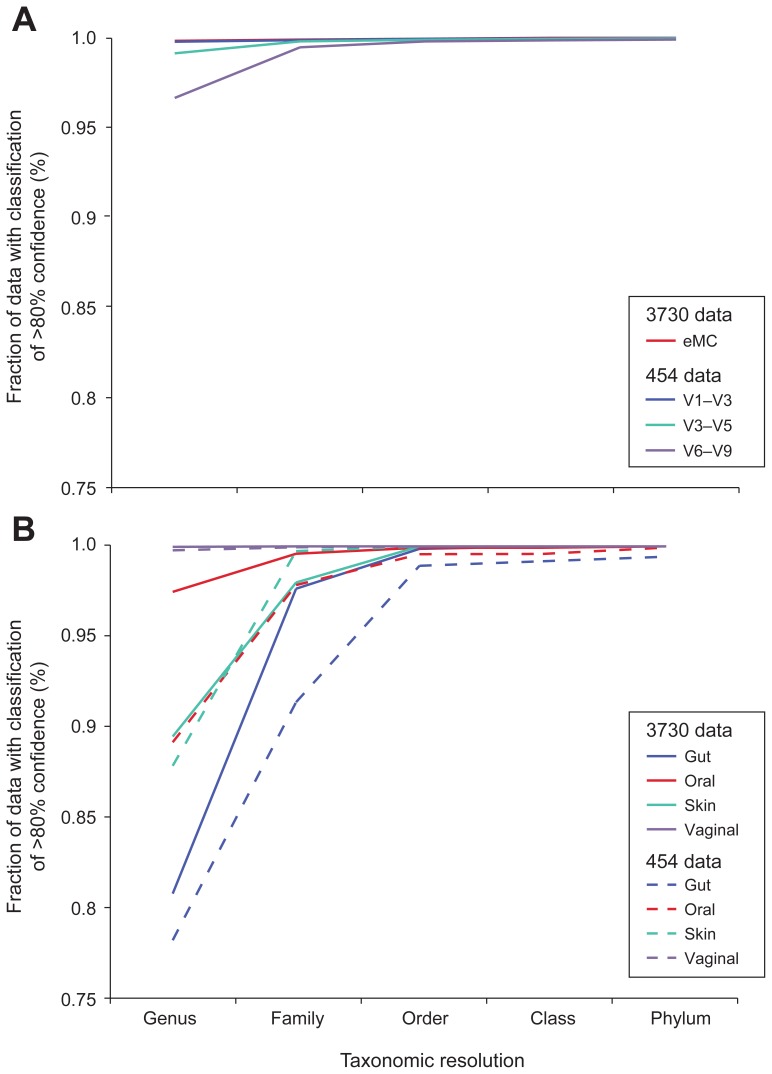
Taxonomic utility of 16S sequence data varies by technology, 16S window, and sample type. The fraction of successfully classified 3730 and 454 sequences obtained from the MC (A) and clinical samples representing four major body regions (B) is plotted at different taxonomic levels from genus to phylum. Classification was performed on quality and chimera-filtered sequences and considered to be successful if the RDP Classifier result had a confidence score above 80%. In panel B, 454 results include only window V3–V5.

**Figure 9:**
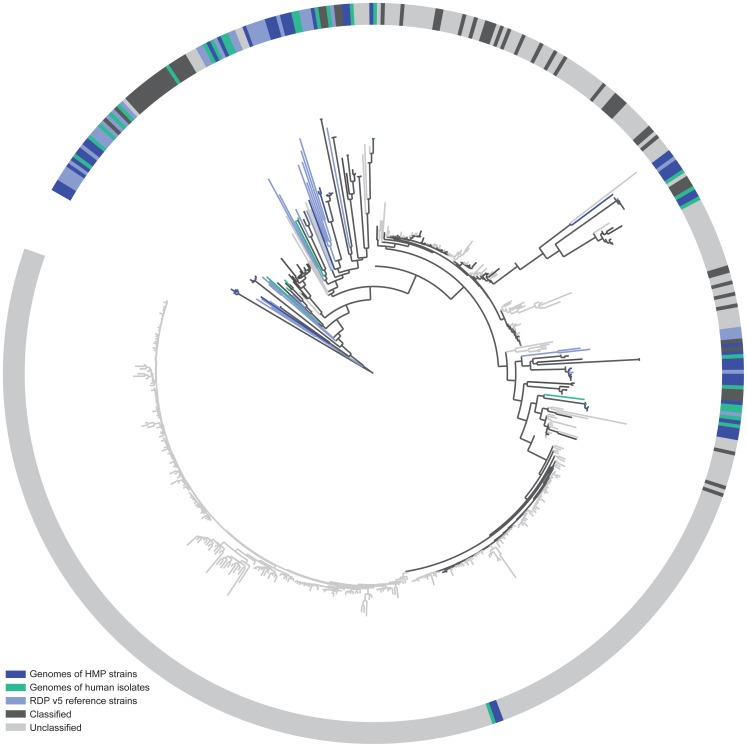
The *Lachnospiraceae* 16S diversity observed in stool samples is greater than from known reference resources. A phylogenetic tree constructed with 16S sequences from RDP’s training set (light blue, n = 34), publicly available genomes from human isolates (green, n = 26), publicly available HMP genomes (dark blue, n = 44), and sequences from aggregate stool samples that could be classified at the genus level (dark grey, n = 63) and that remained unclassified at the genus level (light grey, n = 408).

We compared the relative taxonomic “classifiability” of 16S data from the MC and each clinical sample and, consistent with what we observed previously, all non-chimeric data from the MC exhibited >95% genus level classifiability and 100% at the order level. Among these data, the 3730 sequences and the 454 reads from V1–V3 exhibited the greatest classifiability and the 454 V6–V9 reads the lowest (Fig. 8A). In classifying both 3730 and 454 data from clinical samples, we noted a reduced classification success for data from stool, oral and skin samples relative to that seen with sequences from the MC, while most data from vaginal samples were classifiable (Fig. 8B). We examined the data from our stool samples in greater detail to better understand why only ∼80% of these sequences classified at the genus level. We found that the majority of unclassified sequences fell into two families, *Ruminococcaceae* and *Lachnospiraceae*. Phylogenetic analysis of the *Lachnospiraceae* reads indicated that these sequences likely represented bona fide organisms that were distantly related to the *Lachnospiraceae* represented in the 16S reference set used in our classification regime (Fig. 9). Thus, expansion of the 16S reference sets, particularly with respect to these families, will likely improve the performance of classification methods.

**Figure 10:**
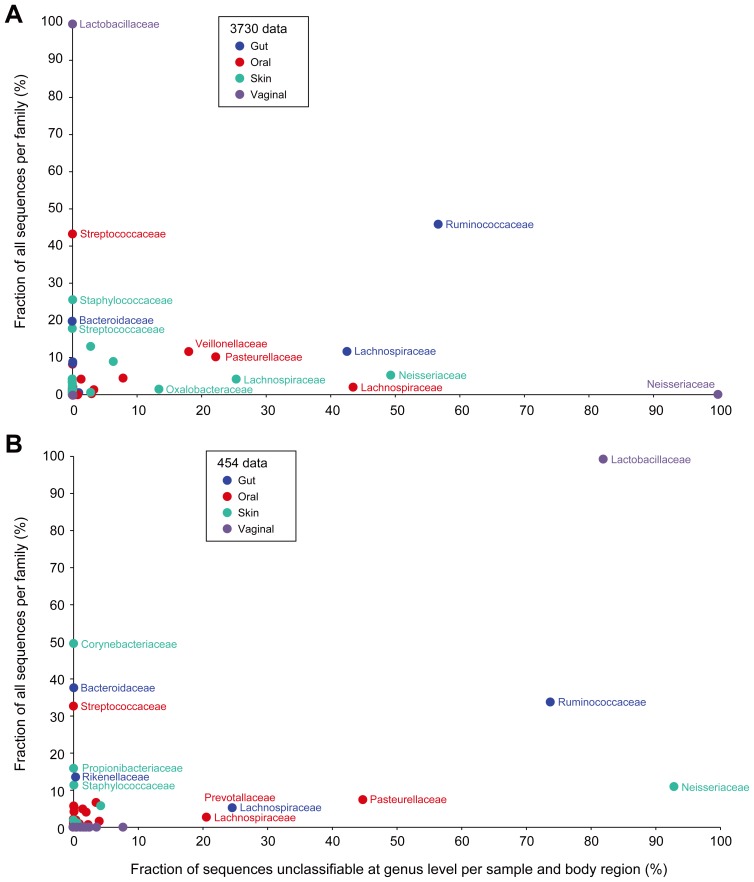
Classifiability of 16S sequence data is differentially impacted by sequencing technology, taxonomic family and body region. For each of the HMP body regions, the relationship between the average frequency of a given bacterial family (y-axis) versus the contribution of these families to the unclassifiability issue (x-axis) is plotted for (B) 3730 and (C) 454. Only window V3–V5 is presented in 454 results. Classification was performed on quality- and chimera-filtered sequences and classifications assigned only if the RDP Classifier result had a confidence score above 80%.

Although 3730-derived sequences from our clinical samples were generally more successfully classified than the shorter 454 reads, the difference was modest for stool, skin and vaginal samples, corresponding to just a few percent. The exception, however, was the data from oral samples where the 3730 sequences demonstrated 10% greater classifiability than the shorter 454 windows. Previously, it was shown that smaller 16S windows generally negatively impacted classification success [25]. We observed this most clearly with the vaginal community (Fig. 10), where the 454-generated sequences belonging to the family *Lactobacillaceae* were more difficult to classify at the genus level compared to those generated using 3730. However, the number of reads affected was low and, therefore, did not contribute greatly to differences in our ability to classify data generated by these two technologies (Fig. 8B). A family of organisms, *Pasteurellaceae*, present at low abundance, explained the difference between the two platforms in the ability to classify reads generated from the oral community (Figs. 10 and 8B).

### Species Richness

**Figure 11:**
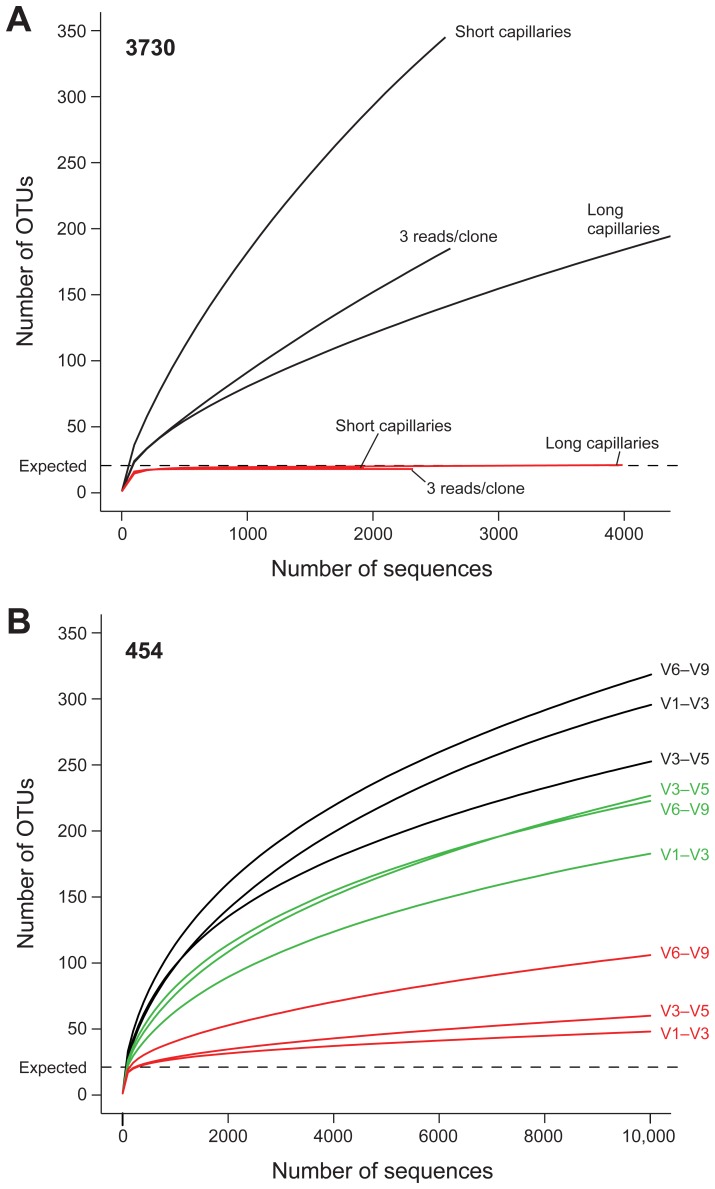
Improved estimation of community diversity after quality filtering and chimera checking, as evaluated by rarefaction analysis. The number of observed OTUs in the MC is shown as the function of the number of 3730 (A) and 454 (B) sequences, before filtering (black), after quality filtering (green, 454 only), and after combined quality and chimera filtering (red). Rarefaction curves were generated using mothur, with an OTU defined at 97% similarity. (A) For 3730, separate lines show the rarefaction curves for the three different sequencing approaches. (B) For 454, rarefaction curves for the three 16S windows spanning the variable regions V1–V3, V3–V5, and V6–V9 are shown separately, and analysis was performed on a random 10,000-sequence subset from each sample.

Classification-based methods can oversimplify or miss diversity not represented in the reference taxonomy. Alternatively, evaluation of diversity within a sample by clustering sequences into operational taxonomic units (OTUs) that are defined by sequence similarity thresholds can provide greater resolution. We calculated OTUs at 97% similarity in the MC data in which 21 species were expected to cluster into 18 OTUs (Figs. 11A and 11B, dashed line). Initial clustering of 3730 and 454 data yielded diversity estimates that greatly exceeded the expected (Figs. 11A and 11B, solid black lines).

The 3730 sequencer capillary length had a profound effect on estimates of diversity. Longer capillaries resulted in more accurate, but still greatly inflated, diversity estimates. Three 454 windows yielded approximately similarly inflated diversity estimates (Figs. 11A and 11B, black lines). Chimeric sequences and sequence errors can inflate estimates and standard practice is to filter read data prior to clustering. We filtered reads using simple quality criteria and removed chimeras with CSlayer and Wigeon [18] (see Bioinformatic Methods). For 3730 MC data, quality filtering excluded 10% of the total reads and subsequent chimera filtering excluded an additional 5.4% for a total of 15.4% of reads being excluded. For 454, quality filtering excluded 20.4% of the total reads and subsequent chimera filtering excluded an additional 16.1% for a total of 36.5% of reads excluded. After filtering, 18 OTUs were correctly estimated from 3730 data and estimates from 454 windows were significantly reduced to within a few fold of expected (Figs. 11A and 11B, red lines). Thus, based on assessment of a community of known diversity, both platforms could approximate true diversity.

## Discussion

The scope of the HMP, to profile the microbiome of 300 individuals at numerous body sites over a prolonged period of time and at multiple research sites, imposed a significant obligation to define aspects of data production and data quality that contribute to the consistency, accuracy and utility of the data generated. In the course of establishing protocols (Protocols S1 and S2), we evaluated the performance and error characteristics of each technology, and the relationship of sequence error to the utility of 16S rDNA regions for classification- and OTU-based analysis of community structure.

Here, we generated data using the conventional, long-read 3730 platform and the shorter-read, higher-throughput 454 platform. Unlike reference genome sequencing, where assembly of individual reads produces high-quality consensus sequence, each individual 454 read or assembled 3730 read pair stands separately without the benefit of error correction or removal of anomalous reads by consensus methods. A key facet of the work presented was using a known control, the MC, which allowed us to directly characterize the features contributing to erroneous interpretation of sequence data, and explore simple filters that could in turn be applied to clinical samples of unknown composition.

The primary goal of the HMP, however, is to compare communities within different samples and both 3730 and 454 sequencing will suffice for this purpose. The tremendous cost advantage of 454, because it permits characterization of more samples at greater read depth, cemented its selection as the platform of choice for the 16S production phase of the HMP. Although Illumina sequencing platforms were not a viable option at the time these data were generated, similar advantages of cost and depth are currently driving rapid adoption of Illumina-based approaches. Recent work has reported on the applicability of Illumina sequencing to 16S rDNA studies [26]. Because, much of the existing reference-quality data for 16S rDNA were generated using Sanger sequencing methods, we believe direct comparisons between next generation platforms and 3730 are useful to assess the quality of new experimental data and reference standards.

We noted the quality of 3730 data varied. The highest quality data were generated using longer capillaries, with assembled, overlapping reads from each end of the near full-length amplicon. For the shorter reads generated by 454 sequencing, the highest quality data were generated using the V1–V3 and V3–V5 windows. When applied to the MC, the V6–V9 window performed poorest, producing the greatest diversity overestimations in the OTU analysis, lower classifiability and higher error rates. V6–V9 also performed less consistently in inter-center comparisons. When applied to identical clinical samples, the V1–V3 and V3–V5 windows produced different representations of the communities and varied in their sensitivity to different organisms. For example, V1–V3 failed to adequately amplify members of the *Bifidobacteriaceae* family (data not shown). Both windows allow differentiation of samples from varying body sites and individuals. It is not clear that either window is fundamentally more accurate than the other across all potential applications. However, V3–V5 did described our MC more accurately than the other windows. The use of multiple windows may, collectively, give the most complete description of a community. However, like others, we caution against comingling V6–V9 data with other data types [27].

We clearly illustrate that sequence artifacts can result in mis-classification of reads. It was recently demonstrated that identical chimeric artifacts can be reproduced across independent experiments and were abundant in data from both 3730 and 454 sequencing [18], suggesting that observation of a novel sequence in multiple samples may not sufficiently confirm the existence of novel taxa. The MC sequence artifacts we observed impacted the interpretation of species diversity and created the appearance of taxa not present. Applied to clinical samples, 454 data could be classified at approximately the same frequency as 3730 data; the oral data was an exception, where a higher proportion of the 3730 data was classifiable. Some of the sample-dependent variation in successful taxonomic classification of these data reflected non-uniform reliability of the underlying taxonomic scheme applied. These difficulties will be ameliorated as additional reference sequences become available and taxonomic schemes receive continued curation.

Sequencing MC 16S rDNA demonstrated that both 3730- and 454-produced data overestimate species richness to a similar extent. After filtering sequences with an excessive number of low-quality bases and chimeric sequences, the near full-length, assembled 3730 sequences produced data that accurately reflected species richness while the shorter 454 reads still yielded spurious OTUs.

The informatic processing of read data is a significant component of 16S rDNA work. We applied simple filtering metrics in combination with recently developed chimera detection algorithms [18]. This approach dramatically reduced the percentage of misclassified reads, and significantly lowered or eliminated spurious OTUs from both 3730 and 454 data. Other groups have also explored optimization of denoising, filtering and clustering approaches for pre-processing data [28]–[31]. In this work, we have confirmed and complemented many of these observations and present a large and rich data set, encompassing a highly defined MC, multiple clinical samples from diverse body habitats, and data generated from multiple centers applying both unique and consolidated protocols.

The results presented, along with the works of others, demonstrate that all facets of data production and data processing can generate artifacts that bias the representation of community membership. This presents an opportunity for the research community to investigate how to better consolidate and advance approaches for metagenomic studies. As additional sequencing technologies are applied to community metagenomics, it will be critical to benchmark and standardize against defined references so that the research community can leverage the combined data sets efficiently and effectively to obtain greater insights.

## Materials and Methods

### Molecular Methods

#### Mock community composition

The organisms for the mock community (MC) include a variety of different genera commonly found on or within the human body. The MC composition has been described elsewhere [18] and additional data is available on the HMP Data Analysis and Coordination Center website (http://www.hmpdacc.org/). Genomic DNA from each organism was prepared individually and the DNAs were then mixed, based on 16S rRNA gene copy number, to create the MC. The organisms included were *Acinetobacter baumannii* ATCC 17978, *Actinomyces odontolyticus* ATCC 17982, *Bacillus cereus* ATCC 10987, *Bacteroides vulgatus* ATCC 8482, *Clostridium beijerinckii* ATCC 51743, *Deinococcus radiodurans* DSM 20539 (ATCC 13939), *Enterococcus faecalis* ATCC 47077, *Escherichia coli* ATCC 700926, *Helicobacter pylori* ATCC 700392, *Lactobacillus gasseri* DSM 20243 (ATCC 33323), *Listeria monocytogenes* ATCC BAA-679, *Methanobrevibacter smithii* ATCC 35061, *Neisseria meningitidis* ATCC BAA-335, *Propionibacterium acnes* DSM1 6379, *Pseudomonas aeruginosa* ATCC 47085, *Rhodobacter sphaeroides* ATCC 17023, *Staphylococcus aureus* ATCC BAA-1718, *Staphylococcus epidermidis* ATCC 12228, *Streptococcus agalactiae* ATCC BAA-611, *Streptococcus mutans* ATCC 700610, and *Streptococcus pneumoniae* ATCC BAA-334. *Candida albicans* ATCC MYA-2876 was included as a negative control but limited to only 1,000 18S copies (calculated) per µl.

### Clinical Samples

Clinical samples were collected non-invasively at Baylor College of Medicine in Houston, TX and Washington University in St. Louis, MO. IRB approval for clinical samples used in this study were granted from Baylor College of Medicine (IRB Approval #22895) and The Human Research Protection Office of Washington University in St. Louis (IRB Approval #08-0754). The collecting institutions obtained written, informed consent from all participants. DNA from clinical samples was provided to the sequencing centers. Information describing the collection and extraction of DNA from clinical samples, documents representing the consent forms used and supplemental study information is available on the HMP Data Analysis and Coordination Center website (http://www.hmpdacc.org/).

### Amplification and Cloning of Full-length 16S rRNA Genes for 3730 Sequencing

Samples were amplified and sequenced according to the “HMP 3730 16S Protocol Version 1.1″ (Protocol S1) using established primer sequences [32], [33]. The protocol is available on the HMP Data Analysis and Coordination Center website (http://www.hmpdacc.org/).

### Amplification and 454 Sequencing of Targeted 16S rRNA Gene Variable Regions

Samples were amplified and sequenced according to the “HMP 454 16S Protocol Version 4.2″ (Protocol S2). The protocol is available on the HMP Data Analysis and Coordination Center website (http://www.hmpdacc.org/). Amplification primers were designed with FLX Titanium adapters (A adapter sequence: 5′ CCATCTCATCCCTGCGTGTCTCCGACTCAG 3′; B adapter sequence: 5′ CCTATCCCCTGTGTGCCTTGGCAGTCTCAG 3′) and a sample barcode sequence where applicable. Forward primers contained the B adapter and the reverse primers contained the A adapter. Specific barcoded primer sequences can be found in Tables S3, S4, S5.

### Bioinformatic Methods

#### Processing and quality filtering of raw sequence data

Default processing of 3730 16S rRNA sequences: Sequences derived from a single clone were assembled using AmosCmp16Spipeline [18], which incorporates filtering to exclude non-16S reads, and trimming of amplification primers sequences and low-quality ends of reads. Assembled sequences were removed from the analysis if they were <1100 nt or if more than 10% of the bases had a Phred quality score below 20. ‘N’ characters were inserted at the gap position when reads did not overlap according to the estimated gap size. Sequences were removed from analysis if the gap size was larger than 1% of the total assembled length. Prior to analysis, bases with a Phred score below 20 were replaced with an ‘N’. Variants of the default processing pipeline that were evaluated include different approaches for base calling (KB base calling versus Phred), trimming (LUCY), and assembly.

Default processing of 454 16S rRNA sequences: Sequences were processed using mothur v.1.6.0 [34]. Sequences were removed from the analysis if they were <200 nt or >600 nt), had a read quality score <25, contained ambiguous characters, had a non-exact barcode match, or contained more than four mismatches to the reverse primer sequence (i.e., 534R, 926R, and 1492R). After assignment of sequences to samples based on barcode matches, the barcode and primer sequences were trimmed and reads were oriented such that all sequences begin with the 5-prime end according to standard sense strand conventions.

For detection of chimeric sequences, all 16S rDNA sequences were aligned using NAST-iEr [18] to obtain a fixed-width alignment, and subjected to CSlayer and WigeoN [18]. Note that for all these tools, we used the original implementation made available at http://microbiomeutil.sourceforge.net/. Sequences that were flagged as chimeric by either of the two methods were removed from further analysis.

#### Taxonomic assignment of 16S sequences/reads

Naïve Bayesian classification: RDP classifier (v2.2) software was used to classify the sequences according to the taxonomy proposed by Garrity et al. [35] and maintained at the Ribosomal Database Project (RDP 10 database, version 6). We used a cutoff for the RDP classifier confidence score of 80%.

BLAST-based classification: The identities of the 16S sequences were determined by creating a BLAST database of the genomes representing all organisms included in the mock community and then performing a BLASTn alignment (97% identity and 90% coverage) of the 16S sequences to the database. These results were parsed to obtain the top hit for each sequence and the top hits were counted to obtain the number of sequences matching each genome.

#### Determination of MC composition from Illumina WGS sequences

The MC was subjected to WGS sequencing on the Illumina platform to generate 240,935,824 101-nt reads. The Burrows-Wheeler Aligner (BWA) [36] was used to align read fastq files to a fasta file of the bacterial reference genomes of MC members resulting in 192,543,566 mapped reads. A sorted BAM and pileup file were created with SAMtools software suite [37]. For each organism in the MC, breadth and depth of coverage was determined as follows. Breadth: the number of reference bases with coverage greater than 0 was divided by the genome’s size. Depth: the sum of the coverages at each base was divided by the number of bases covered. All but one organism had >95% breadth of coverage (S. aureus, 93.74%). The relative depth of coverage was used to derive the expected composition of the MC.

#### Analysis of variation

PCoA was performed on the frequencies of identified genera, with unclassified reads excluded from the analysis. The covariance matrix of the data was used to construct the eigenvectors [38]. For each of the two reported axes, we performed ANOVA using center, sample, and center-by-sample effects. Using the sums-of-squares, we estimated the relative contributions of each covariate (effect), and then, weighting by the contribution of each axis, obtained the total variation. Spearman’s rank correlation coefficient was used to assess correlations between intra-center, inter-center, and inter-sample pairwise similarities.

#### Species richness estimation

Rarefaction curves were generated with mothur [34] using the average-neighbor algorithm to assign sequences into operational taxonomic units with a distance level of 3%. For the 454 data sets, a random subset of 10,000 reads was selected.

#### Error profiling

For each of the organisms in the mock community, the available 16S reads were subjected to AMOScmp [39] to perform a reference-based multiple sequence alignment (MSA). From the MSA, the distribution of nucleotides for each position was tallied and a root mean square deviation (RMSD) value was computed between each position and the reference 16S copy using ANDES [40]. A visual comparison of the different 16S windows and sequencing technologies was plotted on a position-by-position basis.

#### Data availability

Data generated for this work can be accessed at http://www.ncbi.nlm.nih.gov/genomeprj/48489.

The NCBI bioproject ID numbers corresponding to figures within this work are as follows. Figures 2 & 3 = 48471 (3730) and 48341 (454), Figure 8A  = 48471 (3730) and 48341 (454), Figure 8B  = 34129 (3730) and 48335 (454), Figure 11A  = 48471, Figure 11B  = 48341, Table 1 = 48471 and 48341. The NCBI bioproject ID for the WGS data used to quantify the MC is 48341.

## Supporting Information

Protocol S1 HMP 3730 16S Protocol Version 1.1.
(PDF)

Protocol S2 HMP 454 16S Protocol Version 4.3.
(PDF)

Table S1 Read Counts for 454 data in Figures 2 & 3.
(DOCX)

Table S2 Read Counts for 3730 data in Figures 2 & 3.
(DOCX)

Table S3 Broad Barcoded Oligos (V1–V3).
(DOCX)

Table S4 Broad Barcoded Oligos (V3–V5).
(DOCX)

Table S5 Broad Barcoded Oligos (V6–V9).
(DOCX)
