# Supplementary material for: Evaluation of 16S rDNA-Based Community Profiling for Human Microbiome Research
Source: PLoS One. 2012 Jun 13;7(6):e39315. doi: 10.1371/journal.pone.0039315 (PMC3374619; doi:10.1371/journal.pone.0039315)
Supplement: Protocol S1 — HMP 3730 16S Protocol Version 1.1. (PDF) [file pone.0039315.s001.pdf]

HMP 3730 16S Protocol Version 1.1

PURPOSE: This protocol describes the protocol for clone-based clinical sample sequencing on the ABI3730 platform

SOURCE OF SAMPLES: DNA provided by Baylor or Washington University clinical sites

PRODUCTION SPECIFICATIONS:

1. **Notes**
    a. Three PCR reactions (technical replicates) are produced for each sample. These replicates are to be pooled *prior* to gel purification
    b. Elution of final product from the gel purification step is in 35µl of PCR Grade Water
    c. A positive control will be included along with the negative control for each set of core mixes set up to be documented by capturing a digital image
    d. The use of alternative conditions can/should be tried if PCR products are not obtained using the primary protocol. These are listed in Part III: Alternative Amplification Conditions
    e. Optimal Electroporator settings may depend on the equipment being used
    f. **Take all reasonable precautions to prevent contamination throughout this process**. This includes:
        i. Setting up PCR reactions in a designated clean PCR area (with a laminar flow hood equipped with UV light).
        ii. Wiping down the PCR workspace with 10% bleach followed by dH20 and exposing to UV after each use.
        iii. Wearing gloves and disposable lab coats at all times when working in the designated PCR area.

2. **PCR Reactions (**Three technical replicate PCR rxns should be performed for each sample)

    a. **Primers**:
    27f-MP 5' AGRGTTTGATCMTGGCTCAG 3'
    1492r-MP 5' TACGGYTACCTTGTTAYGACTT 3'
    All quantifications are completed using a NanoDrop 1000

    b. **PCR mix per sample:**

PCR Water (MO Bio Certified DNA-Free #17000)	1.90ul
2X Pre-Mix D, E or F (Epicentre Fail Safe PCR System)*	5.00ul
1.2uM Primer 27f MP	1.00ul
1.2uM Primer 1492r-MP	1.00ul
Platinum Taq DNA Polymerase High Fidelity(5U/ul) (Invitrogen #11304)	0.10ul
DNA template (1µl)	1.00ul
	10.00ul

* Epicentre Fail Safe PCR System Pre-Mixes: 100 mM Tris-HCl (pH 8.3,22°C), 100 mM KCl, 400 µM of each dNTP. The concentrations of MgCl2 (3-7 mM) and Fail-Safe PCR Enhancer (0-8X) vary with the individual mixes. (http://www.epibio.com/item.asp?id=294)
Currently we have been using Buffer "E."

3. **Controls (to be set up with each core mix)**
   - **a.** To confirm absence of contaminants, prepare a control reaction following above recipe, replacing 1.0ul DNA template with 1.0ul PCR Grade Water. This negative control should be set up with each core mix
   - **b.** To confirm the efficacy of the reagents and reaction conditions, prepare a positive control (e.g. gDNA from an organism amplifiable with the primers being used for PCR). The positive control should be set up with each core mix.
   - **c.** To document the controls 10% of the reaction (1 µl) should be run on an agarose gel along with sample reactions and preserved as a digital image

4. **PCR Cycling :**
   96°C 5min. 1X
   94°C 30sec. }
   57°C 1min. } 30 cycles
   72°C 2min. }
   72°C 7min. 1X
   4°C Hold

5. **Tailing Reaction:**
   - **a.** After amplification place tubes on ice and add 1 U of Taq DNA polymerase, recombinant (Invitrogen SKU# 10342) per tube.
   - **b.** Mix well
   - **c.** Incubate at 72°C for 8-10 minutes (do not cycle)

6. **Check PCR Products of Samples and Controls**
   - **a.** Run 1.0µl of each reaction including positive and negative controls on an agarose gel (e.g. 1.2% Egel (Invitrogen, SKU # G5018-01))
   - **b.** Use 1KB ladder as marker (e.g. Invitrogen, SKU# 15615)
   - **c.** Document the gel by photo/digital image

7. **Gel Purification**
   - **a.** Prior to gel purification, pool all three PCR technical replicates representing one sample into one reaction for gel purification
   - **b.** Prepare a preparative 1.2% agarose gel w/ EtBr (0.01µg/ml final concentration) and load dye (e.g., Invitrogen BlueJuice™ Gel Loading Buffer (10X) SKU# 10816-015 (bromophenol blue) at a 1X concentration (one part loading buffer to nine parts sample).
   - **c.** Run gel at ~5-6 v-cm ~half the length of the gel. On a dark reader (Clare Chemical Research, Transilluminator DR-45M transilluminator) excise bands using clean razor blade.

- **d.** Continue clean up of PCR product with QIAquick Gel Extraction Kit (Qiagen, Cat# 28704). Follow manufacturer protocol: http://www1.qiagen.com/literature/handbooks/literature.aspx?id=1000252
- **e.** Excised bands typically weigh between ~ 100-150mg. We omit the isopropanol wash. Elutions are done with PCR grade water.
- **f.** Use 35µl PCR grade water for elution. Wait ~3minutes after adding water for elution before the final spin.
- **g.** If products are dilute they can be concentrated by reducing the volume of water using a speed vac.

8. 16S rDNA Cloning

Technical replicate cloning reactions can be set up for each sample as needed to produce sufficient clones for sequencing

TOPO TA Cloning® Cloning Kit (pCR®4-TOPO® Invitrogen, cat # K4580)
http://tools.invitrogen.com/content/sfs/manuals/topotaseq_man.pdf

Typical TOPO TA cloning reaction:

16s PCR product (~75ng)	X ul
Salt solution (diluted 1:4 for electroporation)	1.0 ul
pCR®4-TOPO vector	1.0 ul
dH20	Y ul
	6.0 ul

X = 0.5-4ul PCR product
Y = 6-(2+X) volume of H20

- **a.** incubate reaction @ 22C for 30min
- **b.** electroporate for transformation (e.g. Bio Rad Gene Pulser II Electroporation System- electroporator settings: 1.75 kV, 200 ohms, 25uF. These settings may need to be changed depending on the electroporator being used) with electrocompetent *E.coli* (TOP10) cells and 2ul of TOPO rxn as directed
- **c.** recover cells with 250 µl S.O.C. medium
- **d.** shake solution for 1hr @ 37C
- **e.** (JCVI: after shaking/incubation, 160 µl 50% glycerol is added (460 µl total))
- **f.** plate on pre-warmed LB agar w/ 50 µg/ml kanamycin
- **g.** For testing JCVI plates two plates - one with 50 µl transformation plated and one with 5 µl transformation plated w/ 20 µl SOC
- **h.** incubate @ 37C ~16hrs (Typical Titers are from ~50- 150 CFU/µl)
- **i.** Prep for sequencing with default sequencing protocols used at each center

9. Sequencing primers:

M13 F and M13R as given in cloning manual

10. Alternative Amplification Conditions

If the initial PCR reaction does not produce an amplification product or produces a product that is insufficient for further processing the following steps may be taken in an effort to acquire a product.

<u>All changes to PCR protocols should be tracked.</u>

- **a.** Repeat default PCR protocol if still unsuccessful proceed to step 2
- **b.** Increase the amount of template in the PCR reaction and increase amount of HiFi Taq polymerase
- **c.** Use 2.5ul template/0.3µl HiFi Taq
- **d.** Use 5ul template/0.3µl HiFi Taq
- **e.** Other options suggested are:
    - i. Alternative Taq Polymerase
    - ii. Different Epicentre Buffer
    - iii. Increase the number of cycles
