# Supplementary material for: Evaluation of 16S rDNA-Based Community Profiling for Human Microbiome Research
Source: PLoS One. 2012 Jun 13;7(6):e39315. doi: 10.1371/journal.pone.0039315 (PMC3374619; doi:10.1371/journal.pone.0039315)
Supplement: Protocol S2 — HMP 454 16S Protocol Version 4.3. (PDF) [file pone.0039315.s002.pdf]

HMP Data Generation 16S 454 Protocol Version 4.2

PURPOSE: This protocol describes the protocol for the clinical sample sequencing using barcoded primers for 16S variable regions V1-3 and V3-5.

SOURCE OF SAMPLES: DNA provided by Baylor or Washington University clinical sites

PRODUCTION SPECIFICATIONS:

1. No more than one sample per 16S region per barcode will be sequenced per machine run.

2. All amplification pools will include one negative control (water) reaction. Centers should attempt to rotate the primer pair used for the negative control so to not always use the same primer pair.

3. All amplification pools will include one positive control reaction. Centers should attempt to rotate the primer pair used for the positive control so to not always use the same primer pair. The choice of positive control template is at the discretion of the sequencing center.

4. To reduce the potential for primer contamination, working stocks for all primer pairs should be tested for contamination by using each primer pair in an amplification reaction that contains no template. No amplified product should be observed when reactions are examined by electrophoresis on an agarose gel. If working stocks are stamped in batches across numerous aliquot plates, each plate should provide less than one weeks' worth of reactions (e.g. one to five uses). A sample plate from each stamped batch should be tested for contamination.

5. Sequencing centers will quantify the samples received using a fluorescent based assay and record this information.

6. Two attempts will be made to amplify each sample. The attempts will differ in the amount of template used:
   a. **Attempt 1:** 2 μl of template
   b. **Attempt 2:** 5 μl of template
   c. After two attempts the sample can be considered a failure and no further work needs to be completed. However, it is up to the discretion of the center to make further attempts. It is also up to the discretion of the center as whether or not to attempt sequencing of weakly amplifying samples

7. 5,000 reads should be *attempted* for each amplicon from a HMP donor sample

8. Amplicons that produce fewer than 3,000 reads passing QC ("good reads") can be sequenced a second time to reach the deliverable of 3,000 reads passing QC. A center can choose to use the same amplicon, or produce a new amplicon for sequencing.

9. If the minimum number of reads (3,000 passing QC) from an amplicon has not been achieved after two sequencing attempts, no further sequencing needs to be completed.

10. Image capture and signal processing should use the most current version of the fragment processing software (V 2.3 as July 2010) as the default

11. Passing QC metrics for reads are:
    a. > 300 nt (raw read)
    b. Minimum of 300 Q20 bases

12. Technical replication between centers- 2% of HMP donor samples will be sequenced at two centers

13. A sample spreadsheet containing metadata and library construction information as set up by the DACC will be completed by all centers and will accompany the submission of the *.sff files.

PRODUCTION PROTOCOL:

1. REAGENTS

**Materials/Equipment**	Vendor	Catalog Number
AccuPrime™ Taq DNA Polymerase High Fidelity	Invitrogen	12346-086
Forward and Reverse Primers premixed	Operon	custom order
96 well thermocyler plate		
clear adhesive plate seals		
DNAse/RNAse free water	-	-
Thermo Cycler	-	-
Vortex	-	-
Pipettes	-	-
Aerosol resistant pipette tips		
MinElute PCR Purification Kit	Qiagen	28004
Ampure (SPRI) Beads (60mL kit)	Agencourt	A29152
1x low TE, pH 8.0		
Quant-IT ds DNA Assay, high sensitivity	Invitrogen/Molecular Probes	Q33120

2. ADDITIONAL DOCUMENTATION

Quant-iT ds DNA Assay protocol (manufacturer's specifications)
SybrGreen Assay protocol (manufacturer's specifications)
poolingCalculator.xls
MinElute PCR Purification Kit Manual

3. METHOD

The PCR will be carried out using AccuPrime Taq High Fidelity. It is not necessary to setup this reaction on ice, however it is recommended.

- **PCR Primer Setup** Set up of 10uM primer plates (combining barcoded A primer with non-barcoded B primer- see appendix below for primer & tag sequences):
    - 1:10 dilution of the 100uM stocks:
    - For each variable region, set up a working primer plate with 90ul of 1x low TE
    - Add 5ul of each barcoded primer A from 100uM plate to corresponding well position in 10uM dilution plate.
    - Add 5ul of the 100uM of corresponding B adapter to each well of the 96-well plate (final concentration 10uM primer pair).
    - Mix by pipetting up and down.
    - Working concentration of 4uM (2uM each primer)
        - Dilute the 10uM primers 1:2.5 in 1x low TE (add 150ul of TE to each well of the 10uM primer stock plate and mix

- Primers can be stamped out into multiple single use primer plates and store at -20°C until ready to use.

3.1. PCR Setup - Mastermix

3.1.1. MasterMix contains the following amounts per sample:

13.85uL	RNAse/DNAse free water
2uL	10X AccuPrime PCR Buffer II
0.15uL	Accuprime Taq Hifi

16uL	Total Volume of master mix

3.1.2. Multiply all volumes above by the amount of reactions needed plus 10%.
3.1.3. Combine reagents in a 2mL micro centrifuge tube and vortex to mix completely. If more than 100 reactions are needed a 15mL tube should be used.
3.1.4. Using an automated pipette transfer 16uL of master mix into individual wells in the 96 well reaction plate.
3.1.5. Cover plate and spin in a centrifuge at 2000rpm to collect sample at the bottom of the wells.

3.2. PCR Setup

3.2.1. Transfer 2uL DNA sample to their respective reaction wells (See 1.1.1.2.)
3.2.2. Transfer 2uL of barcoded primers from primer plate to corresponding wells in 96 well PCR plate.
3.2.3. Securely seal with clear adhesive plate seal and vortex plate vigorously.
3.2.4. Spin briefly at 2000 rpm in a centrifuge.
3.2.5. Place in thermo cycler and cycle as follows:

95°C 2 min
95°C 20 sec }
50 or 56°C* 30 sec } 30 cycles
72°C 5min }
4° forever
*** 56ºC for V3-1, 50ºC for V5-3**

3.2.6. Clean PCR products using Agencourt AmPure Beads
3.2.6.1. Use Agencourt protocol: 1.8x volume beads (36ul beads) – follow manufacturer's specifications.
3.2.7. Elute beads with 25ul 1x low TE, pH 8.0 and transfer to new 96 well plate.

3.3. PCR Gel Analysis – (E-gel alternative using 1ul of PCR product - faster) - we will know from the Quantification step below if we have product so this step is actually optional.

3.3.1. In a new reaction plate add 1uL PCR product to 1uL 6X loading dye
3.3.2. Cover, vortex to mix, briefly centrifuge to collect sample at the bottom of the well.
3.3.3. Prepare a 1% agarose 1X TAE gel with EtBr.

3.3.4. Load samples and run approximately 1 hour at 100V.
3.3.5. Capture gel image on gel-doc and retain for analysis.

3.4. PCR Product Quantification

3.4.1. Quantify PCR product using SYBR-Green Quantification or Quant-IT ds DNA high sensitivity assay according to the manufacturer's specifications.

3.5. PCR Pooling

3.5.1. Using values from the SYBR Green or Quant-IT quantification, calculate pooling amounts using the poolingCalculator.xls or according to the following formula:
Amount (uL) of each sample = ((vol/2)*(min)) / sampleconc
where:
Vol = total volume of each sample
Min = concentration in ng/ul of the sample with the lowest concentration
Sampleconc = concentration in ng/uL of target sample

3.5.2. Pool samples using a minimum transfer volume of 1uL. If less than 1uL is called for, a dilution must be made. If using the poolingCalculator.xls this will be accounted for.
3.5.3. Using a Qiagen minElute column, purify the pool according to the manufacturer's protocol.

(The Broad normalizes by converting all concentrations to molecules/ul. Determine which sample has the lowest concentration and then dilute all other samples to the same concentration. Pool equal volume of each (5-10ul) sample and then concentrate using a Qiagen MinElute column (elution with 30uL, 1x low TE, pH 8.0).

3.6. Sample Transfer for 454 Library Completion

3.6.1. Proceed directly to the qPCR library step.
3.6.2. **Optional:** Enter emPCR using ¼ the recommended primer concentration to avoid too many molecules amplified on bead. *Higher primer concentrations may result in high signal intensities during run lead to higher mixed reads and shorter read lengths. Image software updates may reduce or eliminate this concern.*

ADDITIONAL PROTOCOL INFORMATION

BROAD INSTITUTE PRIMER SEQUENCES INCLUDING TAGS
454 barcode sequences were designed by the Broad Institute (Pablo Alvarez and Will Brockman) between the A adapter and primer specific sequence (see picture below). Barcoded primer sets have been tested by the Broad Institute.

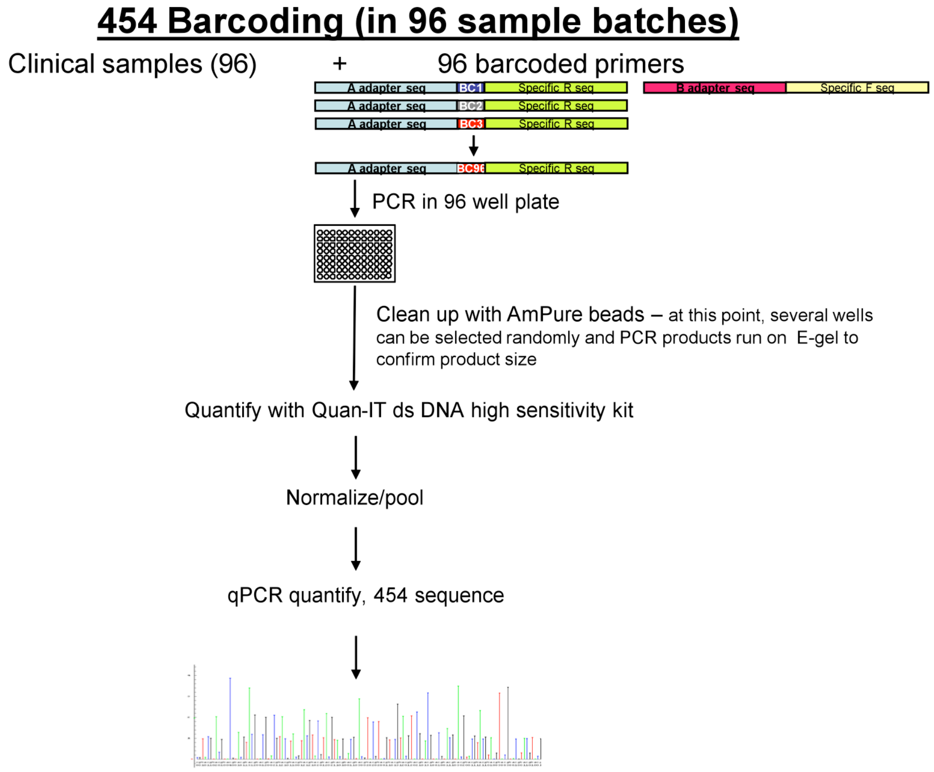

454 Protocol Figure 1: Depiction of generalized workflow for 454 sequencing at HMP sequencing centers.
