# Supplementary material for: Evaluation of 16S rDNA-Based Community Profiling for Human Microbiome Research
Source: PLoS One. 2012 Jun 13;7(6):e39315. doi: 10.1371/journal.pone.0039315 (PMC3374619; doi:10.1371/journal.pone.0039315)
Supplement: Table S1 — Read Counts for 454 data in Figures 2 & 3. (DOCX) [file pone.0039315.s003.docx]

| **Table S1: Read Counts for 454 data in Figures 2 & 3** | | | |  |
| --- | --- | --- | --- | --- |
|  |  |  |  | |
| **Center** | **replicate** | **16S window** | **read count** | |
| A | rep1_run1 | V69 | 26918 | |
| A | rep1_run1 | V13 | 32044 | |
| A | rep1_run1 | V35 | 26073 | |
| A | rep1_run2 | V69 | 24786 | |
| A | rep1_run2 | V13 | 31607 | |
| A | rep1_run2 | V35 | 23831 | |
| A | rep2_run1 | V69 | 26078 | |
| A | rep2_run1 | V13 | 34688 | |
| A | rep2_run1 | V35 | 25930 | |
| A | rep2_run2 | V69 | 23948 | |
| A | rep2_run2 | V13 | 33784 | |
| A | rep2_run2 | V35 | 23886 | |
| A | rep3_run1 | V69 | 30040 | |
| A | rep3_run1 | V13 | 36488 | |
| A | rep3_run1 | V35 | 26976 | |
| A | rep3_run2 | V69 | 27484 | |
| A | rep3_run2 | V13 | 35361 | |
| A | rep3_run2 | V35 | 24498 | |
| B | rep1 | V69 | 15707 | |
| B | rep1 | V13 | 26379 | |
| B | rep1 | V35 | 21580 | |
| B | rep2 | V69 | 14908 | |
| B | rep2 | V13 | 29444 | |
| B | rep2 | V35 | 17293 | |
| B | rep3 | V69 | 14255 | |
| B | rep3 | V13 | 30362 | |
| B | rep3 | V35 | 18577 | |
| C | rep1 | V69 | 14309 | |
| C | rep1 | V13 | 21887 | |
| C | rep1 | V35 | 18962 | |
| C | rep2 | V69 | 9953 | |
| C | rep2 | V13 | 13579 | |
| C | rep2 | V35 | 15478 | |
| C | rep3 | V69 | 10078 | |
| C | rep3 | V13 | 22172 | |
| C | rep3 | V35 | 16142 | |
| D | rep1_run1 | V69 | 9683 | |
| D | rep1_run1 | V13 | 8555 | |
| D | rep1_run1 | V35 | 10854 | |
| D | rep1_run2 | V69 | 6266 | |
| D | rep1_run2 | V13 | 8129 | |
| D | rep1_run2 | V35 | 7345 | |
| D | rep2_run1 | V69 | 11011 | |
| D | rep2_run1 | V13 | 11127 | |
| D | rep2_run1 | V35 | 17143 | |
| D | rep2_run2 | V69 | 5574 | |
| D | rep2_run2 | V13 | 8152 | |
| D | rep2_run2 | V35 | 12902 | |
| D | rep3_run1 | V69 | 10113 | |
| D | rep3_run1 | V13 | 11446 | |
| D | rep3_run1 | V35 | 7225 | |
| D | rep3_run2 | V69 | 3443 | |
| D | rep3_run2 | V13 | 7960 | |
| D | rep3_run2 | V35 | 10157 | |
