# Supplementary material for: Evaluation of 16S rDNA-Based Community Profiling for Human Microbiome Research
Source: PLoS One. 2012 Jun 13;7(6):e39315. doi: 10.1371/journal.pone.0039315 (PMC3374619; doi:10.1371/journal.pone.0039315)
Supplement: Table S2 — Read Counts for 3730 data in Figures 2 & 3. (DOCX) [file pone.0039315.s004.docx]

| **Table S2: Read Counts for 3730 data in Figures 2 & 3** | | | |  |
| --- | --- | --- | --- | --- |
|  |  |  |  | |
| **approach** | **replicate** | **read count** |  |  |
| 1 | rep1 | 2327 |  |  |
| 1 | rep2 | 951 |  |  |
| 2 | rep1 | 1341 |  |  |
| 2 | rep2 | 1236 |  |  |
| 3 | rep1 | 1315 |  |  |
| 3 | rep2 | 1301 |  |  |
