# Supplementary material for: Evaluation of 16S rDNA-Based Community Profiling for Human Microbiome Research
Source: PLoS One. 2012 Jun 13;7(6):e39315. doi: 10.1371/journal.pone.0039315 (PMC3374619; doi:10.1371/journal.pone.0039315)
Supplement: Table S3 — Broad Barcoded Oligos (V1–V3). (DOCX) [file pone.0039315.s005.docx]

**Table S3. Barcoded Oligos for V1-V3**

| **Barcoded oligos for V3->V1 directional sequencing.**  **Added the R specific primer sequence at 3' end of barcode on "A" adapter sequence**  **Added the F specific primer sequence at the 3' end of the "B" adapter sequence** | | |
| --- | --- | --- |
|  |  |  |
|  |  | **“B” adapter oligo sequence +27F (AGAGTTTGATCCTGGCTCAG)** |
|  |  | CCTATCCCCTGTGTGCCTTGGCAGTCTCAGAGAGTTTGATCCTGGCTCAG |
|  |  |  |
| **Oligo name** | **Barcode** | **“A” adapter oligo sequence + barcode + 534R (ATTACCGCGGCTGCTGG)** |
| XLR_534R_v2bBar8L | CACGC | CCATCTCATCCCTGCGTGTCTCCGACTCAGCACGCATTACCGCGGCTGCTGG |
| XLR_534R_v2bBar23L | CGCAAC | CCATCTCATCCCTGCGTGTCTCCGACTCAGCGCAACATTACCGCGGCTGCTGG |
| XLR_534R_v2bBar174L | TGAAGC | CCATCTCATCCCTGCGTGTCTCCGACTCAGTGAAGCATTACCGCGGCTGCTGG |
| XLR_534R_v2bBar602L | ACTTGC | CCATCTCATCCCTGCGTGTCTCCGACTCAGACTTGCATTACCGCGGCTGCTGG |
| XLR_534R_v2bBar212L | TCACAC | CCATCTCATCCCTGCGTGTCTCCGACTCAGTCACACATTACCGCGGCTGCTGG |
| XLR_534R_v2bBar25L | CGTGAC | CCATCTCATCCCTGCGTGTCTCCGACTCAGCGTGACATTACCGCGGCTGCTGG |
| XLR_534R_v2bBar622L | ACGCGC | CCATCTCATCCCTGCGTGTCTCCGACTCAGACGCGCATTACCGCGGCTGCTGG |
| XLR_534R_v2bBar72L | CCTCTC | CCATCTCATCCCTGCGTGTCTCCGACTCAGCCTCTCATTACCGCGGCTGCTGG |
| XLR_534R_v2bBar600L | ACTCAC | CCATCTCATCCCTGCGTGTCTCCGACTCAGACTCACATTACCGCGGCTGCTGG |
| XLR_534R_v2bBar559L | AGACAC | CCATCTCATCCCTGCGTGTCTCCGACTCAGAGACACATTACCGCGGCTGCTGG |
| XLR_534R_v2bBar31L | CGACTC | CCATCTCATCCCTGCGTGTCTCCGACTCAGCGACTCATTACCGCGGCTGCTGG |
| XLR_534R_v2bBar551L | AGCTTC | CCATCTCATCCCTGCGTGTCTCCGACTCAGAGCTTCATTACCGCGGCTGCTGG |
| XLR_534R_v2bBar1149L | AAGCCGC | CCATCTCATCCCTGCGTGTCTCCGACTCAGAAGCCGCATTACCGCGGCTGCTGG |
| XLR_534R_v2bBar15L | CAAGAAC | CCATCTCATCCCTGCGTGTCTCCGACTCAGCAAGAACATTACCGCGGCTGCTGG |
| XLR_534R_v2bBar556L | AGTTGGC | CCATCTCATCCCTGCGTGTCTCCGACTCAGAGTTGGCATTACCGCGGCTGCTGG |
| XLR_534R_v2bBar144L | TATCAAC | CCATCTCATCCCTGCGTGTCTCCGACTCAGTATCAACATTACCGCGGCTGCTGG |
| XLR_534R_v2bBar575L | AGGCGGC | CCATCTCATCCCTGCGTGTCTCCGACTCAGAGGCGGCATTACCGCGGCTGCTGG |
| XLR_534R_v2bBar48L | CGGTATC | CCATCTCATCCCTGCGTGTCTCCGACTCAGCGGTATCATTACCGCGGCTGCTGG |
| XLR_534R_v2bBar166L | TGACGAC | CCATCTCATCCCTGCGTGTCTCCGACTCAGTGACGACATTACCGCGGCTGCTGG |
| XLR_534R_v2bBar613L | ACAAGGC | CCATCTCATCCCTGCGTGTCTCCGACTCAGACAAGGCATTACCGCGGCTGCTGG |
| XLR_534R_v2bBar560L | AGACCTC | CCATCTCATCCCTGCGTGTCTCCGACTCAGAGACCTCATTACCGCGGCTGCTGG |
| XLR_534R_v2bBar741L | ATACCAC | CCATCTCATCCCTGCGTGTCTCCGACTCAGATACCACATTACCGCGGCTGCTGG |
| XLR_534R_v2bBar228L | TCGCGGC | CCATCTCATCCCTGCGTGTCTCCGACTCAGTCGCGGCATTACCGCGGCTGCTGG |
| XLR_534R_v2bBar807L | ATCTTAC | CCATCTCATCCCTGCGTGTCTCCGACTCAGATCTTACATTACCGCGGCTGCTGG |
| XLR_534R_v2bBar1273L | AACCAGC | CCATCTCATCCCTGCGTGTCTCCGACTCAGAACCAGCATTACCGCGGCTGCTGG |
| XLR_534R_v2bBar441L | TTCGAGC | CCATCTCATCCCTGCGTGTCTCCGACTCAGTTCGAGCATTACCGCGGCTGCTGG |
| XLR_534R_v2bBar1174L | AAGGTGC | CCATCTCATCCCTGCGTGTCTCCGACTCAGAAGGTGCATTACCGCGGCTGCTGG |
| XLR_534R_v2bBar209L | TCTTGGC | CCATCTCATCCCTGCGTGTCTCCGACTCAGTCTTGGCATTACCGCGGCTGCTGG |
| XLR_534R_v2bBar153L | TAATCTC | CCATCTCATCCCTGCGTGTCTCCGACTCAGTAATCTCATTACCGCGGCTGCTGG |
| XLR_534R_v2bBar213L | TCACCTC | CCATCTCATCCCTGCGTGTCTCCGACTCAGTCACCTCATTACCGCGGCTGCTGG |
| XLR_534R_v2bBar298L | TCCGCTC | CCATCTCATCCCTGCGTGTCTCCGACTCAGTCCGCTCATTACCGCGGCTGCTGG |
| XLR_534R_v2bBar146L | TATTGAC | CCATCTCATCCCTGCGTGTCTCCGACTCAGTATTGACATTACCGCGGCTGCTGG |
| XLR_534R_v2bBar554L | AGTCGAC | CCATCTCATCCCTGCGTGTCTCCGACTCAGAGTCGACATTACCGCGGCTGCTGG |
| XLR_534R_v2bBar646L | ACGGCTC | CCATCTCATCCCTGCGTGTCTCCGACTCAGACGGCTCATTACCGCGGCTGCTGG |
| XLR_534R_v2bBar158L | TGCGTTC | CCATCTCATCCCTGCGTGTCTCCGACTCAGTGCGTTCATTACCGCGGCTGCTGG |
| XLR_534R_v2bBar207L | TCTCGAC | CCATCTCATCCCTGCGTGTCTCCGACTCAGTCTCGACATTACCGCGGCTGCTGG |
| XLR_534R_v2bBar77L | CCAGGAC | CCATCTCATCCCTGCGTGTCTCCGACTCAGCCAGGACATTACCGCGGCTGCTGG |
| XLR_534R_v2bBar601L | ACTCCTC | CCATCTCATCCCTGCGTGTCTCCGACTCAGACTCCTCATTACCGCGGCTGCTGG |
| XLR_534R_v2bBar481L | TTCCTGC | CCATCTCATCCCTGCGTGTCTCCGACTCAGTTCCTGCATTACCGCGGCTGCTGG |
| XLR_534R_v2bBar419L | TTCATAC | CCATCTCATCCCTGCGTGTCTCCGACTCAGTTCATACATTACCGCGGCTGCTGG |
| XLR_534R_v2bBar26L | CGTCGTC | CCATCTCATCCCTGCGTGTCTCCGACTCAGCGTCGTCATTACCGCGGCTGCTGG |
| XLR_534R_v2bBar1172L | AAGGCAC | CCATCTCATCCCTGCGTGTCTCCGACTCAGAAGGCACATTACCGCGGCTGCTGG |
| XLR_534R_v2bBar1210L | AACAACTC | CCATCTCATCCCTGCGTGTCTCCGACTCAGAACAACTCATTACCGCGGCTGCTGG |
| XLR_534R_v2bBar606L | ACACGGAC | CCATCTCATCCCTGCGTGTCTCCGACTCAGACACGGACATTACCGCGGCTGCTGG |
| XLR_534R_v2bBar159L | TGCCGAAC | CCATCTCATCCCTGCGTGTCTCCGACTCAGTGCCGAACATTACCGCGGCTGCTGG |
| XLR_534R_v2bBar147L | TATTCGTC | CCATCTCATCCCTGCGTGTCTCCGACTCAGTATTCGTCATTACCGCGGCTGCTGG |
| XLR_534R_v2bBar141L | TAGGAATC | CCATCTCATCCCTGCGTGTCTCCGACTCAGTAGGAATCATTACCGCGGCTGCTGG |
| XLR_534R_v2bBar119L | CCGGCCAC | CCATCTCATCCCTGCGTGTCTCCGACTCAGCCGGCCACATTACCGCGGCTGCTGG |
| XLR_534R_v2bBar1379L | AATGGTAC | CCATCTCATCCCTGCGTGTCTCCGACTCAGAATGGTACATTACCGCGGCTGCTGG |
| XLR_534R_v2bBar208L | TCTCCGTC | CCATCTCATCCCTGCGTGTCTCCGACTCAGTCTCCGTCATTACCGCGGCTGCTGG |
| XLR_534R_v2bBar1267L | AACCTGGC | CCATCTCATCCCTGCGTGTCTCCGACTCAGAACCTGGCATTACCGCGGCTGCTGG |
| XLR_534R_v2bBar637L | ACGAAGTC | CCATCTCATCCCTGCGTGTCTCCGACTCAGACGAAGTCATTACCGCGGCTGCTGG |
| XLR_534R_v2bBar435L | TTCGTGGC | CCATCTCATCCCTGCGTGTCTCCGACTCAGTTCGTGGCATTACCGCGGCTGCTGG |
| XLR_534R_v2bBar1202L | AACACAAC | CCATCTCATCCCTGCGTGTCTCCGACTCAGAACACAACATTACCGCGGCTGCTGG |
| XLR_534R_v2bBar413L | TTCTTGAC | CCATCTCATCCCTGCGTGTCTCCGACTCAGTTCTTGACATTACCGCGGCTGCTGG |
| XLR_534R_v2bBar289L | TCCAAGTC | CCATCTCATCCCTGCGTGTCTCCGACTCAGTCCAAGTCATTACCGCGGCTGCTGG |
| XLR_534R_v2bBar433L | TTCGCGAC | CCATCTCATCCCTGCGTGTCTCCGACTCAGTTCGCGACATTACCGCGGCTGCTGG |
| XLR_534R_v2bBar121L | CCGGTCGC | CCATCTCATCCCTGCGTGTCTCCGACTCAGCCGGTCGCATTACCGCGGCTGCTGG |
| XLR_534R_v2bBar669L | ACCTGAAC | CCATCTCATCCCTGCGTGTCTCCGACTCAGACCTGAACATTACCGCGGCTGCTGG |
| XLR_534R_v2bBar1156L | AAGAGTTC | CCATCTCATCCCTGCGTGTCTCCGACTCAGAAGAGTTCATTACCGCGGCTGCTGG |
| XLR_534R_v2bBar370L | TTGACAAC | CCATCTCATCCCTGCGTGTCTCCGACTCAGTTGACAACATTACCGCGGCTGCTGG |
| XLR_534R_v2bBar281L | TCCAGAAC | CCATCTCATCCCTGCGTGTCTCCGACTCAGTCCAGAACATTACCGCGGCTGCTGG |
| XLR_534R_v2bBar49L | CGGTCTTC | CCATCTCATCCCTGCGTGTCTCCGACTCAGCGGTCTTCATTACCGCGGCTGCTGG |
| XLR_534R_v2bBar1173L | AAGGCCTC | CCATCTCATCCCTGCGTGTCTCCGACTCAGAAGGCCTCATTACCGCGGCTGCTGG |
| XLR_534R_v2bBar599L | ACTAATTC | CCATCTCATCCCTGCGTGTCTCCGACTCAGACTAATTCATTACCGCGGCTGCTGG |
| XLR_534R_v2bBar167L | TGACCGTC | CCATCTCATCCCTGCGTGTCTCCGACTCAGTGACCGTCATTACCGCGGCTGCTGG |
| XLR_534R_v2bBar161L | TGTCGGAC | CCATCTCATCCCTGCGTGTCTCCGACTCAGTGTCGGACATTACCGCGGCTGCTGG |
| XLR_534R_v2bBar580L | AGGTTGTC | CCATCTCATCCCTGCGTGTCTCCGACTCAGAGGTTGTCATTACCGCGGCTGCTGG |
| XLR_534R_v2bBar629L | ACGAGAAC | CCATCTCATCCCTGCGTGTCTCCGACTCAGACGAGAACATTACCGCGGCTGCTGG |
| XLR_534R_v2bBar184L | TGGTGAAC | CCATCTCATCCCTGCGTGTCTCCGACTCAGTGGTGAACATTACCGCGGCTGCTGG |
| XLR_534R_v2bBar233L | TCGTTGTC | CCATCTCATCCCTGCGTGTCTCCGACTCAGTCGTTGTCATTACCGCGGCTGCTGG |
| XLR_534R_v2bBar364L | TTGTGTTC | CCATCTCATCCCTGCGTGTCTCCGACTCAGTTGTGTTCATTACCGCGGCTGCTGG |
| XLR_534R_v2bBar78L | CCACGGTC | CCATCTCATCCCTGCGTGTCTCCGACTCAGCCACGGTCATTACCGCGGCTGCTGG |
| XLR_534R_v2bBar393L | TTGGAGGC | CCATCTCATCCCTGCGTGTCTCCGACTCAGTTGGAGGCATTACCGCGGCTGCTGG |
| XLR_534R_v2bBar350L | TTATCGGC | CCATCTCATCCCTGCGTGTCTCCGACTCAGTTATCGGCATTACCGCGGCTGCTGG |
| XLR_534R_v2bBar1164L | AAGAAGAC | CCATCTCATCCCTGCGTGTCTCCGACTCAGAAGAAGACATTACCGCGGCTGCTGG |
| XLR_534R_v2bBar1196L | AACTGTTC | CCATCTCATCCCTGCGTGTCTCCGACTCAGAACTGTTCATTACCGCGGCTGCTGG |
| XLR_534R_v2bBar411L | TTCTCAAC | CCATCTCATCCCTGCGTGTCTCCGACTCAGTTCTCAACATTACCGCGGCTGCTGG |
| XLR_534R_v2bBar6L | CTTCCTTC | CCATCTCATCCCTGCGTGTCTCCGACTCAGCTTCCTTCATTACCGCGGCTGCTGG |
| XLR_534R_v2bBar1031L | ATTCGTAC | CCATCTCATCCCTGCGTGTCTCCGACTCAGATTCGTACATTACCGCGGCTGCTGG |
| XLR_534R_v2bBar76L | CCTTCCGC | CCATCTCATCCCTGCGTGTCTCCGACTCAGCCTTCCGCATTACCGCGGCTGCTGG |
| XLR_534R_v2bBar555L | AGTCCGTC | CCATCTCATCCCTGCGTGTCTCCGACTCAGAGTCCGTCATTACCGCGGCTGCTGG |
| XLR_534R_v2bBar378L | TTGAACTC | CCATCTCATCCCTGCGTGTCTCCGACTCAGTTGAACTCATTACCGCGGCTGCTGG |
| XLR_534R_v2bBar1225L | AACGAGGC | CCATCTCATCCCTGCGTGTCTCCGACTCAGAACGAGGCATTACCGCGGCTGCTGG |
| XLR_534R_v2bBar99L | CCGTTCAC | CCATCTCATCCCTGCGTGTCTCCGACTCAGCCGTTCACATTACCGCGGCTGCTGG |
| XLR_534R_v2bBar236L | TCGAGGAAC | CCATCTCATCCCTGCGTGTCTCCGACTCAGTCGAGGAACATTACCGCGGCTGCTGG |
| XLR_534R_v2bBar731L | ACCGGAAGC | CCATCTCATCCCTGCGTGTCTCCGACTCAGACCGGAAGCATTACCGCGGCTGCTGG |
| XLR_534R_v2bBar628L | ACGTTCCAC | CCATCTCATCCCTGCGTGTCTCCGACTCAGACGTTCCACATTACCGCGGCTGCTGG |
| XLR_534R_v2bBar1250L | AACGGAGTC | CCATCTCATCCCTGCGTGTCTCCGACTCAGAACGGAGTCATTACCGCGGCTGCTGG |
| XLR_534R_v2bBar438L | TTCGTTATC | CCATCTCATCCCTGCGTGTCTCCGACTCAGTTCGTTATCATTACCGCGGCTGCTGG |
| XLR_534R_v2bBar693L | ACCGTAATC | CCATCTCATCCCTGCGTGTCTCCGACTCAGACCGTAATCATTACCGCGGCTGCTGG |
| XLR_534R_v2bBar672L | ACCTTGGTC | CCATCTCATCCCTGCGTGTCTCCGACTCAGACCTTGGTCATTACCGCGGCTGCTGG |
| XLR_534R_v2bBar355L | TTAAGATTC | CCATCTCATCCCTGCGTGTCTCCGACTCAGTTAAGATTCATTACCGCGGCTGCTGG |
| XLR_534R_v2bBar187L | TGGTTGGTC | CCATCTCATCCCTGCGTGTCTCCGACTCAGTGGTTGGTCATTACCGCGGCTGCTGG |
| XLR_534R_v2bBar162L | TGTCCGGTC | CCATCTCATCCCTGCGTGTCTCCGACTCAGTGTCCGGTCATTACCGCGGCTGCTGG |
| XLR_534R_v2bBar1292L | AACCGTGTC | CCATCTCATCCCTGCGTGTCTCCGACTCAGAACCGTGTCATTACCGCGGCTGCTGG |
| 27F/534R_000 | CGTGTGACTG | CCATCTCATCCCTGCGTGTCTCCGACTCAGCGTGTGACTGATTACCGCGGCTGCTGG |
| 27F/534R_001 | CAGATACGAC | CCATCTCATCCCTGCGTGTCTCCGACTCAGCAGATACGACATTACCGCGGCTGCTGG |
| 27F/534R_002 | AGCTCGAGCG | CCATCTCATCCCTGCGTGTCTCCGACTCAGAGCTCGAGCGATTACCGCGGCTGCTGG |
| 27F/534R_003 | CTATCGAGAG | CCATCTCATCCCTGCGTGTCTCCGACTCAGCTATCGAGAGATTACCGCGGCTGCTGG |
| 27F/534R_004 | CTGACTATCG | CCATCTCATCCCTGCGTGTCTCCGACTCAGCTGACTATCGATTACCGCGGCTGCTGG |
| 27F/534R_005 | ATATATAGCG | CCATCTCATCCCTGCGTGTCTCCGACTCAGATATATAGCGATTACCGCGGCTGCTGG |
| 27F/534R_006 | CAGTACGATG | CCATCTCATCCCTGCGTGTCTCCGACTCAGCAGTACGATGATTACCGCGGCTGCTGG |
| 27F/534R_007 | ACTCGCTAGC | CCATCTCATCCCTGCGTGTCTCCGACTCAGACTCGCTAGCATTACCGCGGCTGCTGG |
| 27F/534R_008 | TGAGTCTATC | CCATCTCATCCCTGCGTGTCTCCGACTCAGTGAGTCTATCATTACCGCGGCTGCTGG |
| 27F/534R_009 | TAGCACTACT | CCATCTCATCCCTGCGTGTCTCCGACTCAGTAGCACTACTATTACCGCGGCTGCTGG |
| 27F/534R_010 | AGCGTACGTG | CCATCTCATCCCTGCGTGTCTCCGACTCAGAGCGTACGTGATTACCGCGGCTGCTGG |
| 27F/534R_011 | ACTCGTGTAC | CCATCTCATCCCTGCGTGTCTCCGACTCAGACTCGTGTACATTACCGCGGCTGCTGG |
| 27F/534R_012 | TCTACAGTAG | CCATCTCATCCCTGCGTGTCTCCGACTCAGTCTACAGTAGATTACCGCGGCTGCTGG |
| 27F/534R_013 | ACTATACATC | CCATCTCATCCCTGCGTGTCTCCGACTCAGACTATACATCATTACCGCGGCTGCTGG |
| 27F/534R_014 | TGCGCGAGTG | CCATCTCATCCCTGCGTGTCTCCGACTCAGTGCGCGAGTGATTACCGCGGCTGCTGG |
| 27F/534R_015 | TCGCACACGT | CCATCTCATCCCTGCGTGTCTCCGACTCAGTCGCACACGTATTACCGCGGCTGCTGG |
| 27F/534R_016 | AGCTATATCG | CCATCTCATCCCTGCGTGTCTCCGACTCAGAGCTATATCGATTACCGCGGCTGCTGG |
| 27F/534R_017 | ACGATCGTAT | CCATCTCATCCCTGCGTGTCTCCGACTCAGACGATCGTATATTACCGCGGCTGCTGG |
| 27F/534R_018 | TGCATATACG | CCATCTCATCCCTGCGTGTCTCCGACTCAGTGCATATACGATTACCGCGGCTGCTGG |
| 27F/534R_019 | CGAGACACTG | CCATCTCATCCCTGCGTGTCTCCGACTCAGCGAGACACTGATTACCGCGGCTGCTGG |
| 27F/534R_020 | TGTGCGCTAG | CCATCTCATCCCTGCGTGTCTCCGACTCAGTGTGCGCTAGATTACCGCGGCTGCTGG |
| 27F/534R_021 | TCGTCACGCG | CCATCTCATCCCTGCGTGTCTCCGACTCAGTCGTCACGCGATTACCGCGGCTGCTGG |
| 27F/534R_022 | CACTCACTAG | CCATCTCATCCCTGCGTGTCTCCGACTCAGCACTCACTAGATTACCGCGGCTGCTGG |
| 27F/534R_023 | TGTACAGCTC | CCATCTCATCCCTGCGTGTCTCCGACTCAGTGTACAGCTCATTACCGCGGCTGCTGG |
| 27F/534R_024 | CTGTCTGACG | CCATCTCATCCCTGCGTGTCTCCGACTCAGCTGTCTGACGATTACCGCGGCTGCTGG |
| 27F/534R_025 | CACACTCGCG | CCATCTCATCCCTGCGTGTCTCCGACTCAGCACACTCGCGATTACCGCGGCTGCTGG |
| 27F/534R_026 | CGCTCGTCTG | CCATCTCATCCCTGCGTGTCTCCGACTCAGCGCTCGTCTGATTACCGCGGCTGCTGG |
| 27F/534R_027 | AGCGACGTCT | CCATCTCATCCCTGCGTGTCTCCGACTCAGAGCGACGTCTATTACCGCGGCTGCTGG |
| 27F/534R_028 | CTCACGACGC | CCATCTCATCCCTGCGTGTCTCCGACTCAGCTCACGACGCATTACCGCGGCTGCTGG |
| 27F/534R_029 | ATGTCAGTCG | CCATCTCATCCCTGCGTGTCTCCGACTCAGATGTCAGTCGATTACCGCGGCTGCTGG |
| 27F/534R_030 | TCATAGACAC | CCATCTCATCCCTGCGTGTCTCCGACTCAGTCATAGACACATTACCGCGGCTGCTGG |
| 27F/534R_031 | ATGTACGTGT | CCATCTCATCCCTGCGTGTCTCCGACTCAGATGTACGTGTATTACCGCGGCTGCTGG |
| 27F/534R_032 | ATAGCGTGAG | CCATCTCATCCCTGCGTGTCTCCGACTCAGATAGCGTGAGATTACCGCGGCTGCTGG |
| 27F/534R_033 | TCTGTAGCTC | CCATCTCATCCCTGCGTGTCTCCGACTCAGTCTGTAGCTCATTACCGCGGCTGCTGG |
| 27F/534R_034 | TGATATCGTC | CCATCTCATCCCTGCGTGTCTCCGACTCAGTGATATCGTCATTACCGCGGCTGCTGG |
| 27F/534R_035 | TCACTACATG | CCATCTCATCCCTGCGTGTCTCCGACTCAGTCACTACATGATTACCGCGGCTGCTGG |
| 27F/534R_036 | AGATACGCAG | CCATCTCATCCCTGCGTGTCTCCGACTCAGAGATACGCAGATTACCGCGGCTGCTGG |
| 27F/534R_037 | TATGACTGAG | CCATCTCATCCCTGCGTGTCTCCGACTCAGTATGACTGAGATTACCGCGGCTGCTGG |
| 27F/534R_038 | AGCTGACTAG | CCATCTCATCCCTGCGTGTCTCCGACTCAGAGCTGACTAGATTACCGCGGCTGCTGG |
| 27F/534R_039 | CGCTACGCGC | CCATCTCATCCCTGCGTGTCTCCGACTCAGCGCTACGCGCATTACCGCGGCTGCTGG |
| 27F/534R_040 | ACTGAGTGAG | CCATCTCATCCCTGCGTGTCTCCGACTCAGACTGAGTGAGATTACCGCGGCTGCTGG |
| 27F/534R_041 | AGACGCTACT | CCATCTCATCCCTGCGTGTCTCCGACTCAGAGACGCTACTATTACCGCGGCTGCTGG |
| 27F/534R_042 | TATCTAGACG | CCATCTCATCCCTGCGTGTCTCCGACTCAGTATCTAGACGATTACCGCGGCTGCTGG |
| 27F/534R_043 | TCGTACTATC | CCATCTCATCCCTGCGTGTCTCCGACTCAGTCGTACTATCATTACCGCGGCTGCTGG |
| 27F/534R_044 | TACAGTGAGC | CCATCTCATCCCTGCGTGTCTCCGACTCAGTACAGTGAGCATTACCGCGGCTGCTGG |
| 27F/534R_045 | ATCGATAGAC | CCATCTCATCCCTGCGTGTCTCCGACTCAGATCGATAGACATTACCGCGGCTGCTGG |
| 27F/534R_046 | AGCAGAGACG | CCATCTCATCCCTGCGTGTCTCCGACTCAGAGCAGAGACGATTACCGCGGCTGCTGG |
| 27F/534R_047 | CGACGTGCGC | CCATCTCATCCCTGCGTGTCTCCGACTCAGCGACGTGCGCATTACCGCGGCTGCTGG |
| 27F/534R_048 | CACTCTATCG | CCATCTCATCCCTGCGTGTCTCCGACTCAGCACTCTATCGATTACCGCGGCTGCTGG |
| 27F/534R_049 | TGCTCAGACG | CCATCTCATCCCTGCGTGTCTCCGACTCAGTGCTCAGACGATTACCGCGGCTGCTGG |
| 27F/534R_050 | ACGATGCTCG | CCATCTCATCCCTGCGTGTCTCCGACTCAGACGATGCTCGATTACCGCGGCTGCTGG |
| 27F/534R_051 | TCGTAGCACG | CCATCTCATCCCTGCGTGTCTCCGACTCAGTCGTAGCACGATTACCGCGGCTGCTGG |
| 27F/534R_052 | TCGCGCATCG | CCATCTCATCCCTGCGTGTCTCCGACTCAGTCGCGCATCGATTACCGCGGCTGCTGG |
| 27F/534R_053 | TCGACGCTCT | CCATCTCATCCCTGCGTGTCTCCGACTCAGTCGACGCTCTATTACCGCGGCTGCTGG |
| 27F/534R_054 | CGACGCACAG | CCATCTCATCCCTGCGTGTCTCCGACTCAGCGACGCACAGATTACCGCGGCTGCTGG |
| 27F/534R_055 | TGCGTAGACT | CCATCTCATCCCTGCGTGTCTCCGACTCAGTGCGTAGACTATTACCGCGGCTGCTGG |
| 27F/534R_056 | AGTGTACTGC | CCATCTCATCCCTGCGTGTCTCCGACTCAGAGTGTACTGCATTACCGCGGCTGCTGG |
| 27F/534R_057 | CTAGACTCAG | CCATCTCATCCCTGCGTGTCTCCGACTCAGCTAGACTCAGATTACCGCGGCTGCTGG |
| 27F/534R_058 | AGCGCTGTAG | CCATCTCATCCCTGCGTGTCTCCGACTCAGAGCGCTGTAGATTACCGCGGCTGCTGG |
| 27F/534R_059 | TCTCGAGCAG | CCATCTCATCCCTGCGTGTCTCCGACTCAGTCTCGAGCAGATTACCGCGGCTGCTGG |
| 27F/534R_060 | CGAGTCGAGT | CCATCTCATCCCTGCGTGTCTCCGACTCAGCGAGTCGAGTATTACCGCGGCTGCTGG |
| 27F/534R_061 | TAGCTAGTAT | CCATCTCATCCCTGCGTGTCTCCGACTCAGTAGCTAGTATATTACCGCGGCTGCTGG |
| 27F/534R_062 | AGAGTCGCGC | CCATCTCATCCCTGCGTGTCTCCGACTCAGAGAGTCGCGCATTACCGCGGCTGCTGG |
| 27F/534R_063 | CTCGTCAGTC | CCATCTCATCCCTGCGTGTCTCCGACTCAGCTCGTCAGTCATTACCGCGGCTGCTGG |
| 27F/534R_064 | AGTCTAGTCT | CCATCTCATCCCTGCGTGTCTCCGACTCAGAGTCTAGTCTATTACCGCGGCTGCTGG |
| 27F/534R_065 | TGTACTCACT | CCATCTCATCCCTGCGTGTCTCCGACTCAGTGTACTCACTATTACCGCGGCTGCTGG |
| 27F/534R_066 | CTATGTACAG | CCATCTCATCCCTGCGTGTCTCCGACTCAGCTATGTACAGATTACCGCGGCTGCTGG |
| 27F/534R_067 | TCGTGATAGT | CCATCTCATCCCTGCGTGTCTCCGACTCAGTCGTGATAGTATTACCGCGGCTGCTGG |
| 27F/534R_068 | TGTGTACGAG | CCATCTCATCCCTGCGTGTCTCCGACTCAGTGTGTACGAGATTACCGCGGCTGCTGG |
| 27F/534R_069 | ATCTAGTCAC | CCATCTCATCCCTGCGTGTCTCCGACTCAGATCTAGTCACATTACCGCGGCTGCTGG |
| 27F/534R_070 | TATGAGAGTG | CCATCTCATCCCTGCGTGTCTCCGACTCAGTATGAGAGTGATTACCGCGGCTGCTGG |
| 27F/534R_071 | TACTGCTCAG | CCATCTCATCCCTGCGTGTCTCCGACTCAGTACTGCTCAGATTACCGCGGCTGCTGG |
| 27F/534R_072 | CTATACTACT | CCATCTCATCCCTGCGTGTCTCCGACTCAGCTATACTACTATTACCGCGGCTGCTGG |
| 27F/534R_073 | ACAGTGCTAC | CCATCTCATCCCTGCGTGTCTCCGACTCAGACAGTGCTACATTACCGCGGCTGCTGG |
| 27F/534R_074 | AGTATAGAGC | CCATCTCATCCCTGCGTGTCTCCGACTCAGAGTATAGAGCATTACCGCGGCTGCTGG |
| 27F/534R_075 | ACATCGCGAG | CCATCTCATCCCTGCGTGTCTCCGACTCAGACATCGCGAGATTACCGCGGCTGCTGG |
| 27F/534R_076 | ATGACGACTC | CCATCTCATCCCTGCGTGTCTCCGACTCAGATGACGACTCATTACCGCGGCTGCTGG |
| 27F/534R_077 | TGTATGTACT | CCATCTCATCCCTGCGTGTCTCCGACTCAGTGTATGTACTATTACCGCGGCTGCTGG |
| 27F/534R_078 | CGCGAGATAC | CCATCTCATCCCTGCGTGTCTCCGACTCAGCGCGAGATACATTACCGCGGCTGCTGG |
| 27F/534R_079 | CTACAGTGTG | CCATCTCATCCCTGCGTGTCTCCGACTCAGCTACAGTGTGATTACCGCGGCTGCTGG |
| 27F/534R_080 | TATCACGATG | CCATCTCATCCCTGCGTGTCTCCGACTCAGTATCACGATGATTACCGCGGCTGCTGG |
| 27F/534R_081 | TGCTACGTCG | CCATCTCATCCCTGCGTGTCTCCGACTCAGTGCTACGTCGATTACCGCGGCTGCTGG |
| 27F/534R_082 | CGTCACGTGC | CCATCTCATCCCTGCGTGTCTCCGACTCAGCGTCACGTGCATTACCGCGGCTGCTGG |
| 27F/534R_083 | TCAGCACTCG | CCATCTCATCCCTGCGTGTCTCCGACTCAGTCAGCACTCGATTACCGCGGCTGCTGG |
| 27F/534R_084 | CGTGACTGCG | CCATCTCATCCCTGCGTGTCTCCGACTCAGCGTGACTGCGATTACCGCGGCTGCTGG |
| 27F/534R_085 | ACTATAGTAC | CCATCTCATCCCTGCGTGTCTCCGACTCAGACTATAGTACATTACCGCGGCTGCTGG |
| 27F/534R_086 | ATATGTCGTG | CCATCTCATCCCTGCGTGTCTCCGACTCAGATATGTCGTGATTACCGCGGCTGCTGG |
| 27F/534R_087 | TACTAGATGT | CCATCTCATCCCTGCGTGTCTCCGACTCAGTACTAGATGTATTACCGCGGCTGCTGG |
| 27F/534R_088 | TGTCTGTCTC | CCATCTCATCCCTGCGTGTCTCCGACTCAGTGTCTGTCTCATTACCGCGGCTGCTGG |
| 27F/534R_089 | CGTGACGATC | CCATCTCATCCCTGCGTGTCTCCGACTCAGCGTGACGATCATTACCGCGGCTGCTGG |
| 27F/534R_090 | CGCGTGTCAC | CCATCTCATCCCTGCGTGTCTCCGACTCAGCGCGTGTCACATTACCGCGGCTGCTGG |
| 27F/534R_091 | TCACGTATCT | CCATCTCATCCCTGCGTGTCTCCGACTCAGTCACGTATCTATTACCGCGGCTGCTGG |
| 27F/534R_092 | TAGAGACTAG | CCATCTCATCCCTGCGTGTCTCCGACTCAGTAGAGACTAGATTACCGCGGCTGCTGG |
| 27F/534R_093 | TATGCGCGCG | CCATCTCATCCCTGCGTGTCTCCGACTCAGTATGCGCGCGATTACCGCGGCTGCTGG |
| 27F/534R_094 | CATATACACG | CCATCTCATCCCTGCGTGTCTCCGACTCAGCATATACACGATTACCGCGGCTGCTGG |
| 27F/534R_095 | TCGACTCGAT | CCATCTCATCCCTGCGTGTCTCCGACTCAGTCGACTCGATATTACCGCGGCTGCTGG |
| 27F/534R_096 | ACACAGTCGT | CCATCTCATCCCTGCGTGTCTCCGACTCAGACACAGTCGTATTACCGCGGCTGCTGG |
| 27F/534R_097 | AGTACACGTC | CCATCTCATCCCTGCGTGTCTCCGACTCAGAGTACACGTCATTACCGCGGCTGCTGG |
| 27F/534R_098 | TAGCGATGAC | CCATCTCATCCCTGCGTGTCTCCGACTCAGTAGCGATGACATTACCGCGGCTGCTGG |
| 27F/534R_099 | TGCGTATAGC | CCATCTCATCCCTGCGTGTCTCCGACTCAGTGCGTATAGCATTACCGCGGCTGCTGG |
| 27F/534R_100 | CGACGCGATG | CCATCTCATCCCTGCGTGTCTCCGACTCAGCGACGCGATGATTACCGCGGCTGCTGG |
| 27F/534R_101 | ACGCACTGCG | CCATCTCATCCCTGCGTGTCTCCGACTCAGACGCACTGCGATTACCGCGGCTGCTGG |
| 27F/534R_102 | ACTGTGACTC | CCATCTCATCCCTGCGTGTCTCCGACTCAGACTGTGACTCATTACCGCGGCTGCTGG |
| 27F/534R_103 | TGATCGACAG | CCATCTCATCCCTGCGTGTCTCCGACTCAGTGATCGACAGATTACCGCGGCTGCTGG |
| 27F/534R_104 | TAGTATCGAT | CCATCTCATCCCTGCGTGTCTCCGACTCAGTAGTATCGATATTACCGCGGCTGCTGG |
| 27F/534R_105 | TAGACGCATC | CCATCTCATCCCTGCGTGTCTCCGACTCAGTAGACGCATCATTACCGCGGCTGCTGG |
| 27F/534R_106 | TATCGATCTC | CCATCTCATCCCTGCGTGTCTCCGACTCAGTATCGATCTCATTACCGCGGCTGCTGG |
| 27F/534R_107 | TATCAGTCGT | CCATCTCATCCCTGCGTGTCTCCGACTCAGTATCAGTCGTATTACCGCGGCTGCTGG |
| 27F/534R_108 | ACAGCTATAG | CCATCTCATCCCTGCGTGTCTCCGACTCAGACAGCTATAGATTACCGCGGCTGCTGG |
| 27F/534R_109 | CACTCTCGAC | CCATCTCATCCCTGCGTGTCTCCGACTCAGCACTCTCGACATTACCGCGGCTGCTGG |
| 27F/534R_110 | AGCTACTCTG | CCATCTCATCCCTGCGTGTCTCCGACTCAGAGCTACTCTGATTACCGCGGCTGCTGG |
| 27F/534R_111 | ATACGAGAGC | CCATCTCATCCCTGCGTGTCTCCGACTCAGATACGAGAGCATTACCGCGGCTGCTGG |
| 27F/534R_112 | ACGTCGCAGT | CCATCTCATCCCTGCGTGTCTCCGACTCAGACGTCGCAGTATTACCGCGGCTGCTGG |
| 27F/534R_113 | ATGTCGTACT | CCATCTCATCCCTGCGTGTCTCCGACTCAGATGTCGTACTATTACCGCGGCTGCTGG |
| 27F/534R_114 | CATGTACGTC | CCATCTCATCCCTGCGTGTCTCCGACTCAGCATGTACGTCATTACCGCGGCTGCTGG |
| 27F/534R_115 | CACGCGTCTC | CCATCTCATCCCTGCGTGTCTCCGACTCAGCACGCGTCTCATTACCGCGGCTGCTGG |
| 27F/534R_116 | CGCTATCGAG | CCATCTCATCCCTGCGTGTCTCCGACTCAGCGCTATCGAGATTACCGCGGCTGCTGG |
| 27F/534R_117 | ACGACACGAG | CCATCTCATCCCTGCGTGTCTCCGACTCAGACGACACGAGATTACCGCGGCTGCTGG |
| 27F/534R_118 | TGCGCGTCGC | CCATCTCATCCCTGCGTGTCTCCGACTCAGTGCGCGTCGCATTACCGCGGCTGCTGG |
| 27F/534R_119 | TCAGCTCGTG | CCATCTCATCCCTGCGTGTCTCCGACTCAGTCAGCTCGTGATTACCGCGGCTGCTGG |
| 27F/534R_120 | AGACGACTGT | CCATCTCATCCCTGCGTGTCTCCGACTCAGAGACGACTGTATTACCGCGGCTGCTGG |
| 27F/534R_121 | CACAGTATAC | CCATCTCATCCCTGCGTGTCTCCGACTCAGCACAGTATACATTACCGCGGCTGCTGG |
| 27F/534R_122 | ACGTCATCTG | CCATCTCATCCCTGCGTGTCTCCGACTCAGACGTCATCTGATTACCGCGGCTGCTGG |
| 27F/534R_123 | AGACTGTGAG | CCATCTCATCCCTGCGTGTCTCCGACTCAGAGACTGTGAGATTACCGCGGCTGCTGG |
| 27F/534R_124 | TACACATCAC | CCATCTCATCCCTGCGTGTCTCCGACTCAGTACACATCACATTACCGCGGCTGCTGG |
| 27F/534R_125 | ATAGCTCGAC | CCATCTCATCCCTGCGTGTCTCCGACTCAGATAGCTCGACATTACCGCGGCTGCTGG |
