# Supplementary material for: Evaluation of 16S rDNA-Based Community Profiling for Human Microbiome Research
Source: PLoS One. 2012 Jun 13;7(6):e39315. doi: 10.1371/journal.pone.0039315 (PMC3374619; doi:10.1371/journal.pone.0039315)
Supplement: Table S4 — Broad Barcoded Oligos (V3–V5). (DOCX) [file pone.0039315.s006.docx]

**Table S4. Barcoded Oligos for V3-V5**

| **Barcoded oligos for V5->V3 directional sequencing.**  **Added the R specific primer sequence at 3' end of barcode on "A" adapter sequence**  **Added the F specific primer sequence at the 3' end of the "B" adapter sequence** | | |
| --- | --- | --- |
|  |  |  |
|  |  | **"B" adapter oligo sequence +357F (CCTACGGGAGGCAGCAG)** |
|  |  | CCTATCCCCTGTGTGCCTTGGCAGTCTCAGCCTACGGGAGGCAGCAG |
|  |  |  |
| **Oligo name** | **Barcode** | **"A" adapter oligo sequence + barcode +926R (CCGTCAATTCMTTTRAGT)** |
| XLR_926R_v2bBar8L | CACGC | CCATCTCATCCCTGCGTGTCTCCGACTCAGCACGCCCGTCAATTCMTTTRAGT |
| XLR_926R_v2bBar23L | CGCAAC | CCATCTCATCCCTGCGTGTCTCCGACTCAGCGCAACCCGTCAATTCMTTTRAGT |
| XLR_926R_v2bBar174L | TGAAGC | CCATCTCATCCCTGCGTGTCTCCGACTCAGTGAAGCCCGTCAATTCMTTTRAGT |
| XLR_926R_v2bBar602L | ACTTGC | CCATCTCATCCCTGCGTGTCTCCGACTCAGACTTGCCCGTCAATTCMTTTRAGT |
| XLR_926R_v2bBar212L | TCACAC | CCATCTCATCCCTGCGTGTCTCCGACTCAGTCACACCCGTCAATTCMTTTRAGT |
| XLR_926R_v2bBar25L | CGTGAC | CCATCTCATCCCTGCGTGTCTCCGACTCAGCGTGACCCGTCAATTCMTTTRAGT |
| XLR_926R_v2bBar622L | ACGCGC | CCATCTCATCCCTGCGTGTCTCCGACTCAGACGCGCCCGTCAATTCMTTTRAGT |
| XLR_926R_v2bBar72L | CCTCTC | CCATCTCATCCCTGCGTGTCTCCGACTCAGCCTCTCCCGTCAATTCMTTTRAGT |
| XLR_926R_v2bBar600L | ACTCAC | CCATCTCATCCCTGCGTGTCTCCGACTCAGACTCACCCGTCAATTCMTTTRAGT |
| XLR_926R_v2bBar559L | AGACAC | CCATCTCATCCCTGCGTGTCTCCGACTCAGAGACACCCGTCAATTCMTTTRAGT |
| XLR_926R_v2bBar31L | CGACTC | CCATCTCATCCCTGCGTGTCTCCGACTCAGCGACTCCCGTCAATTCMTTTRAGT |
| XLR_926R_v2bBar551L | AGCTTC | CCATCTCATCCCTGCGTGTCTCCGACTCAGAGCTTCCCGTCAATTCMTTTRAGT |
| XLR_926R_v2bBar1149L | AAGCCGC | CCATCTCATCCCTGCGTGTCTCCGACTCAGAAGCCGCCCGTCAATTCMTTTRAGT |
| XLR_926R_v2bBar15L | CAAGAAC | CCATCTCATCCCTGCGTGTCTCCGACTCAGCAAGAACCCGTCAATTCMTTTRAGT |
| XLR_926R_v2bBar556L | AGTTGGC | CCATCTCATCCCTGCGTGTCTCCGACTCAGAGTTGGCCCGTCAATTCMTTTRAGT |
| XLR_926R_v2bBar144L | TATCAAC | CCATCTCATCCCTGCGTGTCTCCGACTCAGTATCAACCCGTCAATTCMTTTRAGT |
| XLR_926R_v2bBar575L | AGGCGGC | CCATCTCATCCCTGCGTGTCTCCGACTCAGAGGCGGCCCGTCAATTCMTTTRAGT |
| XLR_926R_v2bBar48L | CGGTATC | CCATCTCATCCCTGCGTGTCTCCGACTCAGCGGTATCCCGTCAATTCMTTTRAGT |
| XLR_926R_v2bBar166L | TGACGAC | CCATCTCATCCCTGCGTGTCTCCGACTCAGTGACGACCCGTCAATTCMTTTRAGT |
| XLR_926R_v2bBar613L | ACAAGGC | CCATCTCATCCCTGCGTGTCTCCGACTCAGACAAGGCCCGTCAATTCMTTTRAGT |
| XLR_926R_v2bBar560L | AGACCTC | CCATCTCATCCCTGCGTGTCTCCGACTCAGAGACCTCCCGTCAATTCMTTTRAGT |
| XLR_926R_v2bBar741L | ATACCAC | CCATCTCATCCCTGCGTGTCTCCGACTCAGATACCACCCGTCAATTCMTTTRAGT |
| XLR_926R_v2bBar228L | TCGCGGC | CCATCTCATCCCTGCGTGTCTCCGACTCAGTCGCGGCCCGTCAATTCMTTTRAGT |
| XLR_926R_v2bBar807L | ATCTTAC | CCATCTCATCCCTGCGTGTCTCCGACTCAGATCTTACCCGTCAATTCMTTTRAGT |
| XLR_926R_v2bBar1273L | AACCAGC | CCATCTCATCCCTGCGTGTCTCCGACTCAGAACCAGCCCGTCAATTCMTTTRAGT |
| XLR_926R_v2bBar441L | TTCGAGC | CCATCTCATCCCTGCGTGTCTCCGACTCAGTTCGAGCCCGTCAATTCMTTTRAGT |
| XLR_926R_v2bBar1174L | AAGGTGC | CCATCTCATCCCTGCGTGTCTCCGACTCAGAAGGTGCCCGTCAATTCMTTTRAGT |
| XLR_926R_v2bBar209L | TCTTGGC | CCATCTCATCCCTGCGTGTCTCCGACTCAGTCTTGGCCCGTCAATTCMTTTRAGT |
| XLR_926R_v2bBar153L | TAATCTC | CCATCTCATCCCTGCGTGTCTCCGACTCAGTAATCTCCCGTCAATTCMTTTRAGT |
| XLR_926R_v2bBar213L | TCACCTC | CCATCTCATCCCTGCGTGTCTCCGACTCAGTCACCTCCCGTCAATTCMTTTRAGT |
| XLR_926R_v2bBar298L | TCCGCTC | CCATCTCATCCCTGCGTGTCTCCGACTCAGTCCGCTCCCGTCAATTCMTTTRAGT |
| XLR_926R_v2bBar146L | TATTGAC | CCATCTCATCCCTGCGTGTCTCCGACTCAGTATTGACCCGTCAATTCMTTTRAGT |
| XLR_926R_v2bBar554L | AGTCGAC | CCATCTCATCCCTGCGTGTCTCCGACTCAGAGTCGACCCGTCAATTCMTTTRAGT |
| XLR_926R_v2bBar646L | ACGGCTC | CCATCTCATCCCTGCGTGTCTCCGACTCAGACGGCTCCCGTCAATTCMTTTRAGT |
| XLR_926R_v2bBar158L | TGCGTTC | CCATCTCATCCCTGCGTGTCTCCGACTCAGTGCGTTCCCGTCAATTCMTTTRAGT |
| XLR_926R_v2bBar207L | TCTCGAC | CCATCTCATCCCTGCGTGTCTCCGACTCAGTCTCGACCCGTCAATTCMTTTRAGT |
| XLR_926R_v2bBar77L | CCAGGAC | CCATCTCATCCCTGCGTGTCTCCGACTCAGCCAGGACCCGTCAATTCMTTTRAGT |
| XLR_926R_v2bBar601L | ACTCCTC | CCATCTCATCCCTGCGTGTCTCCGACTCAGACTCCTCCCGTCAATTCMTTTRAGT |
| XLR_926R_v2bBar481L | TTCCTGC | CCATCTCATCCCTGCGTGTCTCCGACTCAGTTCCTGCCCGTCAATTCMTTTRAGT |
| XLR_926R_v2bBar419L | TTCATAC | CCATCTCATCCCTGCGTGTCTCCGACTCAGTTCATACCCGTCAATTCMTTTRAGT |
| XLR_926R_v2bBar26L | CGTCGTC | CCATCTCATCCCTGCGTGTCTCCGACTCAGCGTCGTCCCGTCAATTCMTTTRAGT |
| XLR_926R_v2bBar1172L | AAGGCAC | CCATCTCATCCCTGCGTGTCTCCGACTCAGAAGGCACCCGTCAATTCMTTTRAGT |
| XLR_926R_v2bBar1210L | AACAACTC | CCATCTCATCCCTGCGTGTCTCCGACTCAGAACAACTCCCGTCAATTCMTTTRAGT |
| XLR_926R_v2bBar606L | ACACGGAC | CCATCTCATCCCTGCGTGTCTCCGACTCAGACACGGACCCGTCAATTCMTTTRAGT |
| XLR_926R_v2bBar159L | TGCCGAAC | CCATCTCATCCCTGCGTGTCTCCGACTCAGTGCCGAACCCGTCAATTCMTTTRAGT |
| XLR_926R_v2bBar147L | TATTCGTC | CCATCTCATCCCTGCGTGTCTCCGACTCAGTATTCGTCCCGTCAATTCMTTTRAGT |
| XLR_926R_v2bBar141L | TAGGAATC | CCATCTCATCCCTGCGTGTCTCCGACTCAGTAGGAATCCCGTCAATTCMTTTRAGT |
| XLR_926R_v2bBar119L | CCGGCCAC | CCATCTCATCCCTGCGTGTCTCCGACTCAGCCGGCCACCCGTCAATTCMTTTRAGT |
| XLR_926R_v2bBar1379L | AATGGTAC | CCATCTCATCCCTGCGTGTCTCCGACTCAGAATGGTACCCGTCAATTCMTTTRAGT |
| XLR_926R_v2bBar208L | TCTCCGTC | CCATCTCATCCCTGCGTGTCTCCGACTCAGTCTCCGTCCCGTCAATTCMTTTRAGT |
| XLR_926R_v2bBar1267L | AACCTGGC | CCATCTCATCCCTGCGTGTCTCCGACTCAGAACCTGGCCCGTCAATTCMTTTRAGT |
| XLR_926R_v2bBar637L | ACGAAGTC | CCATCTCATCCCTGCGTGTCTCCGACTCAGACGAAGTCCCGTCAATTCMTTTRAGT |
| XLR_926R_v2bBar435L | TTCGTGGC | CCATCTCATCCCTGCGTGTCTCCGACTCAGTTCGTGGCCCGTCAATTCMTTTRAGT |
| XLR_926R_v2bBar1202L | AACACAAC | CCATCTCATCCCTGCGTGTCTCCGACTCAGAACACAACCCGTCAATTCMTTTRAGT |
| XLR_926R_v2bBar413L | TTCTTGAC | CCATCTCATCCCTGCGTGTCTCCGACTCAGTTCTTGACCCGTCAATTCMTTTRAGT |
| XLR_926R_v2bBar289L | TCCAAGTC | CCATCTCATCCCTGCGTGTCTCCGACTCAGTCCAAGTCCCGTCAATTCMTTTRAGT |
| XLR_926R_v2bBar433L | TTCGCGAC | CCATCTCATCCCTGCGTGTCTCCGACTCAGTTCGCGACCCGTCAATTCMTTTRAGT |
| XLR_926R_v2bBar121L | CCGGTCGC | CCATCTCATCCCTGCGTGTCTCCGACTCAGCCGGTCGCCCGTCAATTCMTTTRAGT |
| XLR_926R_v2bBar669L | ACCTGAAC | CCATCTCATCCCTGCGTGTCTCCGACTCAGACCTGAACCCGTCAATTCMTTTRAGT |
| XLR_926R_v2bBar1156L | AAGAGTTC | CCATCTCATCCCTGCGTGTCTCCGACTCAGAAGAGTTCCCGTCAATTCMTTTRAGT |
| XLR_926R_v2bBar370L | TTGACAAC | CCATCTCATCCCTGCGTGTCTCCGACTCAGTTGACAACCCGTCAATTCMTTTRAGT |
| XLR_926R_v2bBar281L | TCCAGAAC | CCATCTCATCCCTGCGTGTCTCCGACTCAGTCCAGAACCCGTCAATTCMTTTRAGT |
| XLR_926R_v2bBar49L | CGGTCTTC | CCATCTCATCCCTGCGTGTCTCCGACTCAGCGGTCTTCCCGTCAATTCMTTTRAGT |
| XLR_926R_v2bBar1173L | AAGGCCTC | CCATCTCATCCCTGCGTGTCTCCGACTCAGAAGGCCTCCCGTCAATTCMTTTRAGT |
| XLR_926R_v2bBar599L | ACTAATTC | CCATCTCATCCCTGCGTGTCTCCGACTCAGACTAATTCCCGTCAATTCMTTTRAGT |
| XLR_926R_v2bBar167L | TGACCGTC | CCATCTCATCCCTGCGTGTCTCCGACTCAGTGACCGTCCCGTCAATTCMTTTRAGT |
| XLR_926R_v2bBar161L | TGTCGGAC | CCATCTCATCCCTGCGTGTCTCCGACTCAGTGTCGGACCCGTCAATTCMTTTRAGT |
| XLR_926R_v2bBar580L | AGGTTGTC | CCATCTCATCCCTGCGTGTCTCCGACTCAGAGGTTGTCCCGTCAATTCMTTTRAGT |
| XLR_926R_v2bBar629L | ACGAGAAC | CCATCTCATCCCTGCGTGTCTCCGACTCAGACGAGAACCCGTCAATTCMTTTRAGT |
| XLR_926R_v2bBar184L | TGGTGAAC | CCATCTCATCCCTGCGTGTCTCCGACTCAGTGGTGAACCCGTCAATTCMTTTRAGT |
| XLR_926R_v2bBar233L | TCGTTGTC | CCATCTCATCCCTGCGTGTCTCCGACTCAGTCGTTGTCCCGTCAATTCMTTTRAGT |
| XLR_926R_v2bBar364L | TTGTGTTC | CCATCTCATCCCTGCGTGTCTCCGACTCAGTTGTGTTCCCGTCAATTCMTTTRAGT |
| XLR_926R_v2bBar78L | CCACGGTC | CCATCTCATCCCTGCGTGTCTCCGACTCAGCCACGGTCCCGTCAATTCMTTTRAGT |
| XLR_926R_v2bBar393L | TTGGAGGC | CCATCTCATCCCTGCGTGTCTCCGACTCAGTTGGAGGCCCGTCAATTCMTTTRAGT |
| XLR_926R_v2bBar350L | TTATCGGC | CCATCTCATCCCTGCGTGTCTCCGACTCAGTTATCGGCCCGTCAATTCMTTTRAGT |
| XLR_926R_v2bBar1164L | AAGAAGAC | CCATCTCATCCCTGCGTGTCTCCGACTCAGAAGAAGACCCGTCAATTCMTTTRAGT |
| XLR_926R_v2bBar1196L | AACTGTTC | CCATCTCATCCCTGCGTGTCTCCGACTCAGAACTGTTCCCGTCAATTCMTTTRAGT |
| XLR_926R_v2bBar411L | TTCTCAAC | CCATCTCATCCCTGCGTGTCTCCGACTCAGTTCTCAACCCGTCAATTCMTTTRAGT |
| XLR_926R_v2bBar6L | CTTCCTTC | CCATCTCATCCCTGCGTGTCTCCGACTCAGCTTCCTTCCCGTCAATTCMTTTRAGT |
| XLR_926R_v2bBar1031L | ATTCGTAC | CCATCTCATCCCTGCGTGTCTCCGACTCAGATTCGTACCCGTCAATTCMTTTRAGT |
| XLR_926R_v2bBar76L | CCTTCCGC | CCATCTCATCCCTGCGTGTCTCCGACTCAGCCTTCCGCCCGTCAATTCMTTTRAGT |
| XLR_926R_v2bBar555L | AGTCCGTC | CCATCTCATCCCTGCGTGTCTCCGACTCAGAGTCCGTCCCGTCAATTCMTTTRAGT |
| XLR_926R_v2bBar378L | TTGAACTC | CCATCTCATCCCTGCGTGTCTCCGACTCAGTTGAACTCCCGTCAATTCMTTTRAGT |
| XLR_926R_v2bBar1225L | AACGAGGC | CCATCTCATCCCTGCGTGTCTCCGACTCAGAACGAGGCCCGTCAATTCMTTTRAGT |
| XLR_926R_v2bBar99L | CCGTTCAC | CCATCTCATCCCTGCGTGTCTCCGACTCAGCCGTTCACCCGTCAATTCMTTTRAGT |
| XLR_926R_v2bBar236L | TCGAGGAAC | CCATCTCATCCCTGCGTGTCTCCGACTCAGTCGAGGAACCCGTCAATTCMTTTRAGT |
| XLR_926R_v2bBar731L | ACCGGAAGC | CCATCTCATCCCTGCGTGTCTCCGACTCAGACCGGAAGCCCGTCAATTCMTTTRAGT |
| XLR_926R_v2bBar628L | ACGTTCCAC | CCATCTCATCCCTGCGTGTCTCCGACTCAGACGTTCCACCCGTCAATTCMTTTRAGT |
| XLR_926R_v2bBar1250L | AACGGAGTC | CCATCTCATCCCTGCGTGTCTCCGACTCAGAACGGAGTCCCGTCAATTCMTTTRAGT |
| XLR_926R_v2bBar438L | TTCGTTATC | CCATCTCATCCCTGCGTGTCTCCGACTCAGTTCGTTATCCCGTCAATTCMTTTRAGT |
| XLR_926R_v2bBar693L | ACCGTAATC | CCATCTCATCCCTGCGTGTCTCCGACTCAGACCGTAATCCCGTCAATTCMTTTRAGT |
| XLR_926R_v2bBar672L | ACCTTGGTC | CCATCTCATCCCTGCGTGTCTCCGACTCAGACCTTGGTCCCGTCAATTCMTTTRAGT |
| XLR_926R_v2bBar355L | TTAAGATTC | CCATCTCATCCCTGCGTGTCTCCGACTCAGTTAAGATTCCCGTCAATTCMTTTRAGT |
| XLR_926R_v2bBar187L | TGGTTGGTC | CCATCTCATCCCTGCGTGTCTCCGACTCAGTGGTTGGTCCCGTCAATTCMTTTRAGT |
| XLR_926R_v2bBar162L | TGTCCGGTC | CCATCTCATCCCTGCGTGTCTCCGACTCAGTGTCCGGTCCCGTCAATTCMTTTRAGT |
| XLR_926R_v2bBar1292L | AACCGTGTC | CCATCTCATCCCTGCGTGTCTCCGACTCAGAACCGTGTCCCGTCAATTCMTTTRAGT |
| 357F/926R_000 | TCATAGACAG | CCATCTCATCCCTGCGTGTCTCCGACTCAGTCATAGACAGCCGTCAATTCMTTTRAGT |
| 357F/926R_001 | TATCACTACG | CCATCTCATCCCTGCGTGTCTCCGACTCAGTATCACTACGCCGTCAATTCMTTTRAGT |
| 357F/926R_002 | AGCGTCAGTA | CCATCTCATCCCTGCGTGTCTCCGACTCAGAGCGTCAGTACCGTCAATTCMTTTRAGT |
| 357F/926R_003 | CTGTACGTAG | CCATCTCATCCCTGCGTGTCTCCGACTCAGCTGTACGTAGCCGTCAATTCMTTTRAGT |
| 357F/926R_004 | AGTCTCTAGA | CCATCTCATCCCTGCGTGTCTCCGACTCAGAGTCTCTAGACCGTCAATTCMTTTRAGT |
| 357F/926R_005 | AGATACACAG | CCATCTCATCCCTGCGTGTCTCCGACTCAGAGATACACAGCCGTCAATTCMTTTRAGT |
| 357F/926R_006 | ACTCTAGTCT | CCATCTCATCCCTGCGTGTCTCCGACTCAGACTCTAGTCTCCGTCAATTCMTTTRAGT |
| 357F/926R_007 | AGTCAGTGTA | CCATCTCATCCCTGCGTGTCTCCGACTCAGAGTCAGTGTACCGTCAATTCMTTTRAGT |
| 357F/926R_008 | CTACGTCTGT | CCATCTCATCCCTGCGTGTCTCCGACTCAGCTACGTCTGTCCGTCAATTCMTTTRAGT |
| 357F/926R_009 | CGACTACGAG | CCATCTCATCCCTGCGTGTCTCCGACTCAGCGACTACGAGCCGTCAATTCMTTTRAGT |
| 357F/926R_010 | TAGCACACTA | CCATCTCATCCCTGCGTGTCTCCGACTCAGTAGCACACTACCGTCAATTCMTTTRAGT |
| 357F/926R_011 | TACGAGTACA | CCATCTCATCCCTGCGTGTCTCCGACTCAGTACGAGTACACCGTCAATTCMTTTRAGT |
| 357F/926R_012 | TGCTACTGAG | CCATCTCATCCCTGCGTGTCTCCGACTCAGTGCTACTGAGCCGTCAATTCMTTTRAGT |
| 357F/926R_013 | CACGATAGCG | CCATCTCATCCCTGCGTGTCTCCGACTCAGCACGATAGCGCCGTCAATTCMTTTRAGT |
| 357F/926R_014 | TATATCGACA | CCATCTCATCCCTGCGTGTCTCCGACTCAGTATATCGACACCGTCAATTCMTTTRAGT |
| 357F/926R_015 | TGTACTACAT | CCATCTCATCCCTGCGTGTCTCCGACTCAGTGTACTACATCCGTCAATTCMTTTRAGT |
| 357F/926R_016 | AGAGCGCGAG | CCATCTCATCCCTGCGTGTCTCCGACTCAGAGAGCGCGAGCCGTCAATTCMTTTRAGT |
| 357F/926R_017 | CGTAGATCGA | CCATCTCATCCCTGCGTGTCTCCGACTCAGCGTAGATCGACCGTCAATTCMTTTRAGT |
| 357F/926R_018 | TGATGACGCG | CCATCTCATCCCTGCGTGTCTCCGACTCAGTGATGACGCGCCGTCAATTCMTTTRAGT |
| 357F/926R_019 | TCTCTCGAGA | CCATCTCATCCCTGCGTGTCTCCGACTCAGTCTCTCGAGACCGTCAATTCMTTTRAGT |
| 357F/926R_020 | TAGTGTAGCG | CCATCTCATCCCTGCGTGTCTCCGACTCAGTAGTGTAGCGCCGTCAATTCMTTTRAGT |
| 357F/926R_021 | TCACGACGTA | CCATCTCATCCCTGCGTGTCTCCGACTCAGTCACGACGTACCGTCAATTCMTTTRAGT |
| 357F/926R_022 | TGTAGAGTAG | CCATCTCATCCCTGCGTGTCTCCGACTCAGTGTAGAGTAGCCGTCAATTCMTTTRAGT |
| 357F/926R_023 | TGCGTACTCA | CCATCTCATCCCTGCGTGTCTCCGACTCAGTGCGTACTCACCGTCAATTCMTTTRAGT |
| 357F/926R_024 | ACGCACACGT | CCATCTCATCCCTGCGTGTCTCCGACTCAGACGCACACGTCCGTCAATTCMTTTRAGT |
| 357F/926R_025 | TGAGTATGAG | CCATCTCATCCCTGCGTGTCTCCGACTCAGTGAGTATGAGCCGTCAATTCMTTTRAGT |
| 357F/926R_026 | TCTATACGCT | CCATCTCATCCCTGCGTGTCTCCGACTCAGTCTATACGCTCCGTCAATTCMTTTRAGT |
| 357F/926R_027 | CAGTGAGACG | CCATCTCATCCCTGCGTGTCTCCGACTCAGCAGTGAGACGCCGTCAATTCMTTTRAGT |
| 357F/926R_028 | CTAGTATGCG | CCATCTCATCCCTGCGTGTCTCCGACTCAGCTAGTATGCGCCGTCAATTCMTTTRAGT |
| 357F/926R_029 | TCTACAGCGT | CCATCTCATCCCTGCGTGTCTCCGACTCAGTCTACAGCGTCCGTCAATTCMTTTRAGT |
| 357F/926R_030 | ATCGCTAGTA | CCATCTCATCCCTGCGTGTCTCCGACTCAGATCGCTAGTACCGTCAATTCMTTTRAGT |
| 357F/926R_031 | AGCAGCTACG | CCATCTCATCCCTGCGTGTCTCCGACTCAGAGCAGCTACGCCGTCAATTCMTTTRAGT |
| 357F/926R_032 | TCGCTATATA | CCATCTCATCCCTGCGTGTCTCCGACTCAGTCGCTATATACCGTCAATTCMTTTRAGT |
| 357F/926R_033 | TCGCTACGCG | CCATCTCATCCCTGCGTGTCTCCGACTCAGTCGCTACGCGCCGTCAATTCMTTTRAGT |
| 357F/926R_034 | TGAGATCTCG | CCATCTCATCCCTGCGTGTCTCCGACTCAGTGAGATCTCGCCGTCAATTCMTTTRAGT |
| 357F/926R_035 | CGTGAGTGCG | CCATCTCATCCCTGCGTGTCTCCGACTCAGCGTGAGTGCGCCGTCAATTCMTTTRAGT |
| 357F/926R_036 | TCGAGCACGT | CCATCTCATCCCTGCGTGTCTCCGACTCAGTCGAGCACGTCCGTCAATTCMTTTRAGT |
| 357F/926R_037 | AGACATATCG | CCATCTCATCCCTGCGTGTCTCCGACTCAGAGACATATCGCCGTCAATTCMTTTRAGT |
| 357F/926R_038 | TCTCGTGTGT | CCATCTCATCCCTGCGTGTCTCCGACTCAGTCTCGTGTGTCCGTCAATTCMTTTRAGT |
| 357F/926R_039 | ATATACGCGT | CCATCTCATCCCTGCGTGTCTCCGACTCAGATATACGCGTCCGTCAATTCMTTTRAGT |
| 357F/926R_040 | ACGTCTCGCT | CCATCTCATCCCTGCGTGTCTCCGACTCAGACGTCTCGCTCCGTCAATTCMTTTRAGT |
| 357F/926R_041 | CGCGCTACGT | CCATCTCATCCCTGCGTGTCTCCGACTCAGCGCGCTACGTCCGTCAATTCMTTTRAGT |
| 357F/926R_042 | AGAGTCACGA | CCATCTCATCCCTGCGTGTCTCCGACTCAGAGAGTCACGACCGTCAATTCMTTTRAGT |
| 357F/926R_043 | TACACGATGT | CCATCTCATCCCTGCGTGTCTCCGACTCAGTACACGATGTCCGTCAATTCMTTTRAGT |
| 357F/926R_044 | CTACATCACG | CCATCTCATCCCTGCGTGTCTCCGACTCAGCTACATCACGCCGTCAATTCMTTTRAGT |
| 357F/926R_045 | ATACTCTATG | CCATCTCATCCCTGCGTGTCTCCGACTCAGATACTCTATGCCGTCAATTCMTTTRAGT |
| 357F/926R_046 | CTCACGCGAG | CCATCTCATCCCTGCGTGTCTCCGACTCAGCTCACGCGAGCCGTCAATTCMTTTRAGT |
| 357F/926R_047 | TCAGTCTCGA | CCATCTCATCCCTGCGTGTCTCCGACTCAGTCAGTCTCGACCGTCAATTCMTTTRAGT |
| 357F/926R_048 | AGAGTACGTG | CCATCTCATCCCTGCGTGTCTCCGACTCAGAGAGTACGTGCCGTCAATTCMTTTRAGT |
| 357F/926R_049 | TGAGACGAGA | CCATCTCATCCCTGCGTGTCTCCGACTCAGTGAGACGAGACCGTCAATTCMTTTRAGT |
| 357F/926R_050 | TATCTGTATA | CCATCTCATCCCTGCGTGTCTCCGACTCAGTATCTGTATACCGTCAATTCMTTTRAGT |
| 357F/926R_051 | TAGCGTGATG | CCATCTCATCCCTGCGTGTCTCCGACTCAGTAGCGTGATGCCGTCAATTCMTTTRAGT |
| 357F/926R_052 | ACTGTATATG | CCATCTCATCCCTGCGTGTCTCCGACTCAGACTGTATATGCCGTCAATTCMTTTRAGT |
| 357F/926R_053 | CATATAGACG | CCATCTCATCCCTGCGTGTCTCCGACTCAGCATATAGACGCCGTCAATTCMTTTRAGT |
| 357F/926R_054 | ACGTATGACT | CCATCTCATCCCTGCGTGTCTCCGACTCAGACGTATGACTCCGTCAATTCMTTTRAGT |
| 357F/926R_055 | TGTCGTAGCA | CCATCTCATCCCTGCGTGTCTCCGACTCAGTGTCGTAGCACCGTCAATTCMTTTRAGT |
| 357F/926R_056 | AGTGACTAGT | CCATCTCATCCCTGCGTGTCTCCGACTCAGAGTGACTAGTCCGTCAATTCMTTTRAGT |
| 357F/926R_057 | TCTATCTAGT | CCATCTCATCCCTGCGTGTCTCCGACTCAGTCTATCTAGTCCGTCAATTCMTTTRAGT |
| 357F/926R_058 | CTAGAGTGTG | CCATCTCATCCCTGCGTGTCTCCGACTCAGCTAGAGTGTGCCGTCAATTCMTTTRAGT |
| 357F/926R_059 | TAGCTACTGT | CCATCTCATCCCTGCGTGTCTCCGACTCAGTAGCTACTGTCCGTCAATTCMTTTRAGT |
| 357F/926R_060 | ATACTGCGTA | CCATCTCATCCCTGCGTGTCTCCGACTCAGATACTGCGTACCGTCAATTCMTTTRAGT |
| 357F/926R_061 | TCAGCGTCTA | CCATCTCATCCCTGCGTGTCTCCGACTCAGTCAGCGTCTACCGTCAATTCMTTTRAGT |
| 357F/926R_062 | CGTACTCAGT | CCATCTCATCCCTGCGTGTCTCCGACTCAGCGTACTCAGTCCGTCAATTCMTTTRAGT |
| 357F/926R_063 | TCGACGAGCT | CCATCTCATCCCTGCGTGTCTCCGACTCAGTCGACGAGCTCCGTCAATTCMTTTRAGT |
| 357F/926R_064 | AGTCGACATG | CCATCTCATCCCTGCGTGTCTCCGACTCAGAGTCGACATGCCGTCAATTCMTTTRAGT |
| 357F/926R_065 | CGTCAGCACG | CCATCTCATCCCTGCGTGTCTCCGACTCAGCGTCAGCACGCCGTCAATTCMTTTRAGT |
| 357F/926R_066 | TCGCTGATAG | CCATCTCATCCCTGCGTGTCTCCGACTCAGTCGCTGATAGCCGTCAATTCMTTTRAGT |
| 357F/926R_067 | ACATACTGTG | CCATCTCATCCCTGCGTGTCTCCGACTCAGACATACTGTGCCGTCAATTCMTTTRAGT |
| 357F/926R_068 | TCATCGAGTG | CCATCTCATCCCTGCGTGTCTCCGACTCAGTCATCGAGTGCCGTCAATTCMTTTRAGT |
| 357F/926R_069 | ACTATATCTA | CCATCTCATCCCTGCGTGTCTCCGACTCAGACTATATCTACCGTCAATTCMTTTRAGT |
| 357F/926R_070 | ACTGTCTGTA | CCATCTCATCCCTGCGTGTCTCCGACTCAGACTGTCTGTACCGTCAATTCMTTTRAGT |
| 357F/926R_071 | CGTGTCGCAG | CCATCTCATCCCTGCGTGTCTCCGACTCAGCGTGTCGCAGCCGTCAATTCMTTTRAGT |
| 357F/926R_072 | CTAGTGCACG | CCATCTCATCCCTGCGTGTCTCCGACTCAGCTAGTGCACGCCGTCAATTCMTTTRAGT |
| 357F/926R_073 | TATACACGCG | CCATCTCATCCCTGCGTGTCTCCGACTCAGTATACACGCGCCGTCAATTCMTTTRAGT |
| 357F/926R_074 | AGTCTAGTAG | CCATCTCATCCCTGCGTGTCTCCGACTCAGAGTCTAGTAGCCGTCAATTCMTTTRAGT |
| 357F/926R_075 | CTCGTGACGT | CCATCTCATCCCTGCGTGTCTCCGACTCAGCTCGTGACGTCCGTCAATTCMTTTRAGT |
| 357F/926R_076 | TACACAGTCA | CCATCTCATCCCTGCGTGTCTCCGACTCAGTACACAGTCACCGTCAATTCMTTTRAGT |
| 357F/926R_077 | CTACAGAGAG | CCATCTCATCCCTGCGTGTCTCCGACTCAGCTACAGAGAGCCGTCAATTCMTTTRAGT |
| 357F/926R_078 | TGACGTGACA | CCATCTCATCCCTGCGTGTCTCCGACTCAGTGACGTGACACCGTCAATTCMTTTRAGT |
| 357F/926R_079 | TCTGTGACAG | CCATCTCATCCCTGCGTGTCTCCGACTCAGTCTGTGACAGCCGTCAATTCMTTTRAGT |
| 357F/926R_080 | TCTGCAGTAG | CCATCTCATCCCTGCGTGTCTCCGACTCAGTCTGCAGTAGCCGTCAATTCMTTTRAGT |
| 357F/926R_081 | TGTACGCACG | CCATCTCATCCCTGCGTGTCTCCGACTCAGTGTACGCACGCCGTCAATTCMTTTRAGT |
| 357F/926R_082 | CTAGCTCGTA | CCATCTCATCCCTGCGTGTCTCCGACTCAGCTAGCTCGTACCGTCAATTCMTTTRAGT |
| 357F/926R_083 | ATACGCACGT | CCATCTCATCCCTGCGTGTCTCCGACTCAGATACGCACGTCCGTCAATTCMTTTRAGT |
| 357F/926R_084 | TGTCTGCTAG | CCATCTCATCCCTGCGTGTCTCCGACTCAGTGTCTGCTAGCCGTCAATTCMTTTRAGT |
| 357F/926R_085 | AGTCGAGCGA | CCATCTCATCCCTGCGTGTCTCCGACTCAGAGTCGAGCGACCGTCAATTCMTTTRAGT |
| 357F/926R_086 | TATGACAGTG | CCATCTCATCCCTGCGTGTCTCCGACTCAGTATGACAGTGCCGTCAATTCMTTTRAGT |
| 357F/926R_087 | ACATAGTAGT | CCATCTCATCCCTGCGTGTCTCCGACTCAGACATAGTAGTCCGTCAATTCMTTTRAGT |
| 357F/926R_088 | TATGATACTA | CCATCTCATCCCTGCGTGTCTCCGACTCAGTATGATACTACCGTCAATTCMTTTRAGT |
| 357F/926R_089 | TCGACGCATA | CCATCTCATCCCTGCGTGTCTCCGACTCAGTCGACGCATACCGTCAATTCMTTTRAGT |
| 357F/926R_090 | ACGCGAGATA | CCATCTCATCCCTGCGTGTCTCCGACTCAGACGCGAGATACCGTCAATTCMTTTRAGT |
| 357F/926R_091 | ACGATGATCG | CCATCTCATCCCTGCGTGTCTCCGACTCAGACGATGATCGCCGTCAATTCMTTTRAGT |
| 357F/926R_092 | ATCGTAGTGT | CCATCTCATCCCTGCGTGTCTCCGACTCAGATCGTAGTGTCCGTCAATTCMTTTRAGT |
| 357F/926R_093 | TATAGCGTCT | CCATCTCATCCCTGCGTGTCTCCGACTCAGTATAGCGTCTCCGTCAATTCMTTTRAGT |
| 357F/926R_094 | ACTCTGTGAG | CCATCTCATCCCTGCGTGTCTCCGACTCAGACTCTGTGAGCCGTCAATTCMTTTRAGT |
| 357F/926R_095 | AGTAGCGTGT | CCATCTCATCCCTGCGTGTCTCCGACTCAGAGTAGCGTGTCCGTCAATTCMTTTRAGT |
| 357F/926R_096 | CGCGACGTGT | CCATCTCATCCCTGCGTGTCTCCGACTCAGCGCGACGTGTCCGTCAATTCMTTTRAGT |
| 357F/926R_097 | CTGTGTAGAG | CCATCTCATCCCTGCGTGTCTCCGACTCAGCTGTGTAGAGCCGTCAATTCMTTTRAGT |
| 357F/926R_098 | CTCTGTCTCG | CCATCTCATCCCTGCGTGTCTCCGACTCAGCTCTGTCTCGCCGTCAATTCMTTTRAGT |
| 357F/926R_099 | AGACGTCTCT | CCATCTCATCCCTGCGTGTCTCCGACTCAGAGACGTCTCTCCGTCAATTCMTTTRAGT |
| 357F/926R_100 | TCGAGAGTCG | CCATCTCATCCCTGCGTGTCTCCGACTCAGTCGAGAGTCGCCGTCAATTCMTTTRAGT |
| 357F/926R_101 | CTCTCGCGTA | CCATCTCATCCCTGCGTGTCTCCGACTCAGCTCTCGCGTACCGTCAATTCMTTTRAGT |
| 357F/926R_102 | ACGTGTACTA | CCATCTCATCCCTGCGTGTCTCCGACTCAGACGTGTACTACCGTCAATTCMTTTRAGT |
| 357F/926R_103 | TGCTGCGTCG | CCATCTCATCCCTGCGTGTCTCCGACTCAGTGCTGCGTCGCCGTCAATTCMTTTRAGT |
| 357F/926R_104 | CATACTACTA | CCATCTCATCCCTGCGTGTCTCCGACTCAGCATACTACTACCGTCAATTCMTTTRAGT |
| 357F/926R_105 | AGTATCTCAG | CCATCTCATCCCTGCGTGTCTCCGACTCAGAGTATCTCAGCCGTCAATTCMTTTRAGT |
| 357F/926R_106 | TACTGCACAG | CCATCTCATCCCTGCGTGTCTCCGACTCAGTACTGCACAGCCGTCAATTCMTTTRAGT |
| 357F/926R_107 | CGCGCACGCG | CCATCTCATCCCTGCGTGTCTCCGACTCAGCGCGCACGCGCCGTCAATTCMTTTRAGT |
| 357F/926R_108 | TCACACTATA | CCATCTCATCCCTGCGTGTCTCCGACTCAGTCACACTATACCGTCAATTCMTTTRAGT |
| 357F/926R_109 | TGACGCGCTA | CCATCTCATCCCTGCGTGTCTCCGACTCAGTGACGCGCTACCGTCAATTCMTTTRAGT |
| 357F/926R_110 | TGTACGTGTG | CCATCTCATCCCTGCGTGTCTCCGACTCAGTGTACGTGTGCCGTCAATTCMTTTRAGT |
| 357F/926R_111 | TCGTGATACA | CCATCTCATCCCTGCGTGTCTCCGACTCAGTCGTGATACACCGTCAATTCMTTTRAGT |
| 357F/926R_112 | ACACTATCAG | CCATCTCATCCCTGCGTGTCTCCGACTCAGACACTATCAGCCGTCAATTCMTTTRAGT |
| 357F/926R_113 | ATCGACGTCA | CCATCTCATCCCTGCGTGTCTCCGACTCAGATCGACGTCACCGTCAATTCMTTTRAGT |
| 357F/926R_114 | TGATCTAGTA | CCATCTCATCCCTGCGTGTCTCCGACTCAGTGATCTAGTACCGTCAATTCMTTTRAGT |
| 357F/926R_115 | ATGACTGTCG | CCATCTCATCCCTGCGTGTCTCCGACTCAGATGACTGTCGCCGTCAATTCMTTTRAGT |
| 357F/926R_116 | ATCGACAGAG | CCATCTCATCCCTGCGTGTCTCCGACTCAGATCGACAGAGCCGTCAATTCMTTTRAGT |
| 357F/926R_117 | AGTATATGTG | CCATCTCATCCCTGCGTGTCTCCGACTCAGAGTATATGTGCCGTCAATTCMTTTRAGT |
| 357F/926R_118 | AGACTGACAG | CCATCTCATCCCTGCGTGTCTCCGACTCAGAGACTGACAGCCGTCAATTCMTTTRAGT |
| 357F/926R_119 | AGCGCGTAGT | CCATCTCATCCCTGCGTGTCTCCGACTCAGAGCGCGTAGTCCGTCAATTCMTTTRAGT |
| 357F/926R_120 | AGACGATGTG | CCATCTCATCCCTGCGTGTCTCCGACTCAGAGACGATGTGCCGTCAATTCMTTTRAGT |
| 357F/926R_121 | TCAGTAGTGA | CCATCTCATCCCTGCGTGTCTCCGACTCAGTCAGTAGTGACCGTCAATTCMTTTRAGT |
| 357F/926R_122 | TGCAGTCGTG | CCATCTCATCCCTGCGTGTCTCCGACTCAGTGCAGTCGTGCCGTCAATTCMTTTRAGT |
| 357F/926R_123 | TGCGTACATG | CCATCTCATCCCTGCGTGTCTCCGACTCAGTGCGTACATGCCGTCAATTCMTTTRAGT |
| 357F/926R_124 | TCAGATGACG | CCATCTCATCCCTGCGTGTCTCCGACTCAGTCAGATGACGCCGTCAATTCMTTTRAGT |
| 357F/926R_125 | AGTGTGAGTG | CCATCTCATCCCTGCGTGTCTCCGACTCAGAGTGTGAGTGCCGTCAATTCMTTTRAGT |
