# Supplementary material for: Evaluation of 16S rDNA-Based Community Profiling for Human Microbiome Research
Source: PLoS One. 2012 Jun 13;7(6):e39315. doi: 10.1371/journal.pone.0039315 (PMC3374619; doi:10.1371/journal.pone.0039315)
Supplement: Table S5 — Broad Barcoded Oligos (V6–V9). (DOCX) [file pone.0039315.s007.docx]

**Supplementary Table S4. Barcoded Oligos for V6-V9**

| **Barcoded oligos for V9->V6 directional sequencing.**  **Added the R specific primer sequence at 3' end of barcode on "A" adapter sequence**  **Added the F specific primer sequence at the 3' end of the "B" adapter sequence** | | |
| --- | --- | --- |
|  |  |  |
|  |  | **"B" adapter oligo sequence + U968F (AACGCGAAGAACCTTAC)** |
|  |  | CCTATCCCCTGTGTGCCTTGGCAGTCTCAGAACGCGAAGAACCTTAC |
|  |  |  |
| **Oligo name** | **Barcode** | **"A" adapter oligo seq. + barcode + 1492R (TACGGYTACCTTGTTAYGACTT)** |
| XLR_1492R_v2bBar8L | CACGC | CCATCTCATCCCTGCGTGTCTCCGACTCAGCACGCTACGGYTACCTTGTTAYGACTT |
| XLR_1492R_v2bBar23L | CGCAAC | CCATCTCATCCCTGCGTGTCTCCGACTCAGCGCAACTACGGYTACCTTGTTAYGACTT |
| XLR_1492R_v2bBar174L | TGAAGC | CCATCTCATCCCTGCGTGTCTCCGACTCAGTGAAGCTACGGYTACCTTGTTAYGACTT |
| XLR_1492R_v2bBar602L | ACTTGC | CCATCTCATCCCTGCGTGTCTCCGACTCAGACTTGCTACGGYTACCTTGTTAYGACTT |
| XLR_1492R_v2bBar212L | TCACAC | CCATCTCATCCCTGCGTGTCTCCGACTCAGTCACACTACGGYTACCTTGTTAYGACTT |
| XLR_1492R_v2bBar25L | CGTGAC | CCATCTCATCCCTGCGTGTCTCCGACTCAGCGTGACTACGGYTACCTTGTTAYGACTT |
| XLR_1492R_v2bBar622L | ACGCGC | CCATCTCATCCCTGCGTGTCTCCGACTCAGACGCGCTACGGYTACCTTGTTAYGACTT |
| XLR_1492R_v2bBar72L | CCTCTC | CCATCTCATCCCTGCGTGTCTCCGACTCAGCCTCTCTACGGYTACCTTGTTAYGACTT |
| XLR_1492R_v2bBar600L | ACTCAC | CCATCTCATCCCTGCGTGTCTCCGACTCAGACTCACTACGGYTACCTTGTTAYGACTT |
| XLR_1492R_v2bBar559L | AGACAC | CCATCTCATCCCTGCGTGTCTCCGACTCAGAGACACTACGGYTACCTTGTTAYGACTT |
| XLR_1492R_v2bBar31L | CGACTC | CCATCTCATCCCTGCGTGTCTCCGACTCAGCGACTCTACGGYTACCTTGTTAYGACTT |
| XLR_1492R_v2bBar551L | AGCTTC | CCATCTCATCCCTGCGTGTCTCCGACTCAGAGCTTCTACGGYTACCTTGTTAYGACTT |
| XLR_1492R_v2bBar1149L | AAGCCGC | CCATCTCATCCCTGCGTGTCTCCGACTCAGAAGCCGCTACGGYTACCTTGTTAYGACTT |
| XLR_1492R_v2bBar15L | CAAGAAC | CCATCTCATCCCTGCGTGTCTCCGACTCAGCAAGAACTACGGYTACCTTGTTAYGACTT |
| XLR_1492R_v2bBar556L | AGTTGGC | CCATCTCATCCCTGCGTGTCTCCGACTCAGAGTTGGCTACGGYTACCTTGTTAYGACTT |
| XLR_1492R_v2bBar144L | TATCAAC | CCATCTCATCCCTGCGTGTCTCCGACTCAGTATCAACTACGGYTACCTTGTTAYGACTT |
| XLR_1492R_v2bBar575L | AGGCGGC | CCATCTCATCCCTGCGTGTCTCCGACTCAGAGGCGGCTACGGYTACCTTGTTAYGACTT |
| XLR_1492R_v2bBar48L | CGGTATC | CCATCTCATCCCTGCGTGTCTCCGACTCAGCGGTATCTACGGYTACCTTGTTAYGACTT |
| XLR_1492R_v2bBar166L | TGACGAC | CCATCTCATCCCTGCGTGTCTCCGACTCAGTGACGACTACGGYTACCTTGTTAYGACTT |
| XLR_1492R_v2bBar613L | ACAAGGC | CCATCTCATCCCTGCGTGTCTCCGACTCAGACAAGGCTACGGYTACCTTGTTAYGACTT |
| XLR_1492R_v2bBar560L | AGACCTC | CCATCTCATCCCTGCGTGTCTCCGACTCAGAGACCTCTACGGYTACCTTGTTAYGACTT |
| XLR_1492R_v2bBar741L | ATACCAC | CCATCTCATCCCTGCGTGTCTCCGACTCAGATACCACTACGGYTACCTTGTTAYGACTT |
| XLR_1492R_v2bBar228L | TCGCGGC | CCATCTCATCCCTGCGTGTCTCCGACTCAGTCGCGGCTACGGYTACCTTGTTAYGACTT |
| XLR_1492R_v2bBar807L | ATCTTAC | CCATCTCATCCCTGCGTGTCTCCGACTCAGATCTTACTACGGYTACCTTGTTAYGACTT |
| XLR_1492R_v2bBar1273L | AACCAGC | CCATCTCATCCCTGCGTGTCTCCGACTCAGAACCAGCTACGGYTACCTTGTTAYGACTT |
| XLR_1492R_v2bBar441L | TTCGAGC | CCATCTCATCCCTGCGTGTCTCCGACTCAGTTCGAGCTACGGYTACCTTGTTAYGACTT |
| XLR_1492R_v2bBar1174L | AAGGTGC | CCATCTCATCCCTGCGTGTCTCCGACTCAGAAGGTGCTACGGYTACCTTGTTAYGACTT |
| XLR_1492R_v2bBar209L | TCTTGGC | CCATCTCATCCCTGCGTGTCTCCGACTCAGTCTTGGCTACGGYTACCTTGTTAYGACTT |
| XLR_1492R_v2bBar153L | TAATCTC | CCATCTCATCCCTGCGTGTCTCCGACTCAGTAATCTCTACGGYTACCTTGTTAYGACTT |
| XLR_1492R_v2bBar213L | TCACCTC | CCATCTCATCCCTGCGTGTCTCCGACTCAGTCACCTCTACGGYTACCTTGTTAYGACTT |
| XLR_1492R_v2bBar298L | TCCGCTC | CCATCTCATCCCTGCGTGTCTCCGACTCAGTCCGCTCTACGGYTACCTTGTTAYGACTT |
| XLR_1492R_v2bBar146L | TATTGAC | CCATCTCATCCCTGCGTGTCTCCGACTCAGTATTGACTACGGYTACCTTGTTAYGACTT |
| XLR_1492R_v2bBar554L | AGTCGAC | CCATCTCATCCCTGCGTGTCTCCGACTCAGAGTCGACTACGGYTACCTTGTTAYGACTT |
| XLR_1492R_v2bBar646L | ACGGCTC | CCATCTCATCCCTGCGTGTCTCCGACTCAGACGGCTCTACGGYTACCTTGTTAYGACTT |
| XLR_1492R_v2bBar158L | TGCGTTC | CCATCTCATCCCTGCGTGTCTCCGACTCAGTGCGTTCTACGGYTACCTTGTTAYGACTT |
| XLR_1492R_v2bBar207L | TCTCGAC | CCATCTCATCCCTGCGTGTCTCCGACTCAGTCTCGACTACGGYTACCTTGTTAYGACTT |
| XLR_1492R_v2bBar77L | CCAGGAC | CCATCTCATCCCTGCGTGTCTCCGACTCAGCCAGGACTACGGYTACCTTGTTAYGACTT |
| XLR_1492R_v2bBar601L | ACTCCTC | CCATCTCATCCCTGCGTGTCTCCGACTCAGACTCCTCTACGGYTACCTTGTTAYGACTT |
| XLR_1492R_v2bBar481L | TTCCTGC | CCATCTCATCCCTGCGTGTCTCCGACTCAGTTCCTGCTACGGYTACCTTGTTAYGACTT |
| XLR_1492R_v2bBar419L | TTCATAC | CCATCTCATCCCTGCGTGTCTCCGACTCAGTTCATACTACGGYTACCTTGTTAYGACTT |
| XLR_1492R_v2bBar26L | CGTCGTC | CCATCTCATCCCTGCGTGTCTCCGACTCAGCGTCGTCTACGGYTACCTTGTTAYGACTT |
| XLR_1492R_v2bBar1172L | AAGGCAC | CCATCTCATCCCTGCGTGTCTCCGACTCAGAAGGCACTACGGYTACCTTGTTAYGACTT |
| XLR_1492R_v2bBar1210L | AACAACTC | CCATCTCATCCCTGCGTGTCTCCGACTCAGAACAACTCTACGGYTACCTTGTTAYGACTT |
| XLR_1492R_v2bBar606L | ACACGGAC | CCATCTCATCCCTGCGTGTCTCCGACTCAGACACGGACTACGGYTACCTTGTTAYGACTT |
| XLR_1492R_v2bBar159L | TGCCGAAC | CCATCTCATCCCTGCGTGTCTCCGACTCAGTGCCGAACTACGGYTACCTTGTTAYGACTT |
| XLR_1492R_v2bBar147L | TATTCGTC | CCATCTCATCCCTGCGTGTCTCCGACTCAGTATTCGTCTACGGYTACCTTGTTAYGACTT |
| XLR_1492R_v2bBar141L | TAGGAATC | CCATCTCATCCCTGCGTGTCTCCGACTCAGTAGGAATCTACGGYTACCTTGTTAYGACTT |
| XLR_1492R_v2bBar119L | CCGGCCAC | CCATCTCATCCCTGCGTGTCTCCGACTCAGCCGGCCACTACGGYTACCTTGTTAYGACTT |
| XLR_1492R_v2bBar1379L | AATGGTAC | CCATCTCATCCCTGCGTGTCTCCGACTCAGAATGGTACTACGGYTACCTTGTTAYGACTT |
| XLR_1492R_v2bBar208L | TCTCCGTC | CCATCTCATCCCTGCGTGTCTCCGACTCAGTCTCCGTCTACGGYTACCTTGTTAYGACTT |
| XLR_1492R_v2bBar1267L | AACCTGGC | CCATCTCATCCCTGCGTGTCTCCGACTCAGAACCTGGCTACGGYTACCTTGTTAYGACTT |
| XLR_1492R_v2bBar637L | ACGAAGTC | CCATCTCATCCCTGCGTGTCTCCGACTCAGACGAAGTCTACGGYTACCTTGTTAYGACTT |
| XLR_1492R_v2bBar435L | TTCGTGGC | CCATCTCATCCCTGCGTGTCTCCGACTCAGTTCGTGGCTACGGYTACCTTGTTAYGACTT |
| XLR_1492R_v2bBar1202L | AACACAAC | CCATCTCATCCCTGCGTGTCTCCGACTCAGAACACAACTACGGYTACCTTGTTAYGACTT |
| XLR_1492R_v2bBar413L | TTCTTGAC | CCATCTCATCCCTGCGTGTCTCCGACTCAGTTCTTGACTACGGYTACCTTGTTAYGACTT |
| XLR_1492R_v2bBar289L | TCCAAGTC | CCATCTCATCCCTGCGTGTCTCCGACTCAGTCCAAGTCTACGGYTACCTTGTTAYGACTT |
| XLR_1492R_v2bBar433L | TTCGCGAC | CCATCTCATCCCTGCGTGTCTCCGACTCAGTTCGCGACTACGGYTACCTTGTTAYGACTT |
| XLR_1492R_v2bBar121L | CCGGTCGC | CCATCTCATCCCTGCGTGTCTCCGACTCAGCCGGTCGCTACGGYTACCTTGTTAYGACTT |
| XLR_1492R_v2bBar669L | ACCTGAAC | CCATCTCATCCCTGCGTGTCTCCGACTCAGACCTGAACTACGGYTACCTTGTTAYGACTT |
| XLR_1492R_v2bBar1156L | AAGAGTTC | CCATCTCATCCCTGCGTGTCTCCGACTCAGAAGAGTTCTACGGYTACCTTGTTAYGACTT |
| XLR_1492R_v2bBar370L | TTGACAAC | CCATCTCATCCCTGCGTGTCTCCGACTCAGTTGACAACTACGGYTACCTTGTTAYGACTT |
| XLR_1492R_v2bBar281L | TCCAGAAC | CCATCTCATCCCTGCGTGTCTCCGACTCAGTCCAGAACTACGGYTACCTTGTTAYGACTT |
| XLR_1492R_v2bBar49L | CGGTCTTC | CCATCTCATCCCTGCGTGTCTCCGACTCAGCGGTCTTCTACGGYTACCTTGTTAYGACTT |
| XLR_1492R_v2bBar1173L | AAGGCCTC | CCATCTCATCCCTGCGTGTCTCCGACTCAGAAGGCCTCTACGGYTACCTTGTTAYGACTT |
| XLR_1492R_v2bBar599L | ACTAATTC | CCATCTCATCCCTGCGTGTCTCCGACTCAGACTAATTCTACGGYTACCTTGTTAYGACTT |
| XLR_1492R_v2bBar167L | TGACCGTC | CCATCTCATCCCTGCGTGTCTCCGACTCAGTGACCGTCTACGGYTACCTTGTTAYGACTT |
| XLR_1492R_v2bBar161L | TGTCGGAC | CCATCTCATCCCTGCGTGTCTCCGACTCAGTGTCGGACTACGGYTACCTTGTTAYGACTT |
| XLR_1492R_v2bBar580L | AGGTTGTC | CCATCTCATCCCTGCGTGTCTCCGACTCAGAGGTTGTCTACGGYTACCTTGTTAYGACTT |
| XLR_1492R_v2bBar629L | ACGAGAAC | CCATCTCATCCCTGCGTGTCTCCGACTCAGACGAGAACTACGGYTACCTTGTTAYGACTT |
| XLR_1492R_v2bBar184L | TGGTGAAC | CCATCTCATCCCTGCGTGTCTCCGACTCAGTGGTGAACTACGGYTACCTTGTTAYGACTT |
| XLR_1492R_v2bBar233L | TCGTTGTC | CCATCTCATCCCTGCGTGTCTCCGACTCAGTCGTTGTCTACGGYTACCTTGTTAYGACTT |
| XLR_1492R_v2bBar364L | TTGTGTTC | CCATCTCATCCCTGCGTGTCTCCGACTCAGTTGTGTTCTACGGYTACCTTGTTAYGACTT |
| XLR_1492R_v2bBar78L | CCACGGTC | CCATCTCATCCCTGCGTGTCTCCGACTCAGCCACGGTCTACGGYTACCTTGTTAYGACTT |
| XLR_1492R_v2bBar393L | TTGGAGGC | CCATCTCATCCCTGCGTGTCTCCGACTCAGTTGGAGGCTACGGYTACCTTGTTAYGACTT |
| XLR_1492R_v2bBar350L | TTATCGGC | CCATCTCATCCCTGCGTGTCTCCGACTCAGTTATCGGCTACGGYTACCTTGTTAYGACTT |
| XLR_1492R_v2bBar1164L | AAGAAGAC | CCATCTCATCCCTGCGTGTCTCCGACTCAGAAGAAGACTACGGYTACCTTGTTAYGACTT |
| XLR_1492R_v2bBar1196L | AACTGTTC | CCATCTCATCCCTGCGTGTCTCCGACTCAGAACTGTTCTACGGYTACCTTGTTAYGACTT |
| XLR_1492R_v2bBar411L | TTCTCAAC | CCATCTCATCCCTGCGTGTCTCCGACTCAGTTCTCAACTACGGYTACCTTGTTAYGACTT |
| XLR_1492R_v2bBar6L | CTTCCTTC | CCATCTCATCCCTGCGTGTCTCCGACTCAGCTTCCTTCTACGGYTACCTTGTTAYGACTT |
| XLR_1492R_v2bBar1031L | ATTCGTAC | CCATCTCATCCCTGCGTGTCTCCGACTCAGATTCGTACTACGGYTACCTTGTTAYGACTT |
| XLR_1492R_v2bBar76L | CCTTCCGC | CCATCTCATCCCTGCGTGTCTCCGACTCAGCCTTCCGCTACGGYTACCTTGTTAYGACTT |
| XLR_1492R_v2bBar555L | AGTCCGTC | CCATCTCATCCCTGCGTGTCTCCGACTCAGAGTCCGTCTACGGYTACCTTGTTAYGACTT |
| XLR_1492R_v2bBar378L | TTGAACTC | CCATCTCATCCCTGCGTGTCTCCGACTCAGTTGAACTCTACGGYTACCTTGTTAYGACTT |
| XLR_1492R_v2bBar1225L | AACGAGGC | CCATCTCATCCCTGCGTGTCTCCGACTCAGAACGAGGCTACGGYTACCTTGTTAYGACTT |
| XLR_1492R_v2bBar99L | CCGTTCAC | CCATCTCATCCCTGCGTGTCTCCGACTCAGCCGTTCACTACGGYTACCTTGTTAYGACTT |
| XLR_1492R_v2bBar236L | TCGAGGAAC | CCATCTCATCCCTGCGTGTCTCCGACTCAGTCGAGGAACTACGGYTACCTTGTTAYGACTT |
| XLR_1492R_v2bBar731L | ACCGGAAGC | CCATCTCATCCCTGCGTGTCTCCGACTCAGACCGGAAGCTACGGYTACCTTGTTAYGACTT |
| XLR_1492R_v2bBar628L | ACGTTCCAC | CCATCTCATCCCTGCGTGTCTCCGACTCAGACGTTCCACTACGGYTACCTTGTTAYGACTT |
| XLR_1492R_v2bBar1250L | AACGGAGTC | CCATCTCATCCCTGCGTGTCTCCGACTCAGAACGGAGTCTACGGYTACCTTGTTAYGACTT |
| XLR_1492R_v2bBar438L | TTCGTTATC | CCATCTCATCCCTGCGTGTCTCCGACTCAGTTCGTTATCTACGGYTACCTTGTTAYGACTT |
| XLR_1492R_v2bBar693L | ACCGTAATC | CCATCTCATCCCTGCGTGTCTCCGACTCAGACCGTAATCTACGGYTACCTTGTTAYGACTT |
| XLR_1492R_v2bBar672L | ACCTTGGTC | CCATCTCATCCCTGCGTGTCTCCGACTCAGACCTTGGTCTACGGYTACCTTGTTAYGACTT |
| XLR_1492R_v2bBar355L | TTAAGATTC | CCATCTCATCCCTGCGTGTCTCCGACTCAGTTAAGATTCTACGGYTACCTTGTTAYGACTT |
| XLR_1492R_v2bBar187L | TGGTTGGTC | CCATCTCATCCCTGCGTGTCTCCGACTCAGTGGTTGGTCTACGGYTACCTTGTTAYGACTT |
| XLR_1492R_v2bBar162L | TGTCCGGTC | CCATCTCATCCCTGCGTGTCTCCGACTCAGTGTCCGGTCTACGGYTACCTTGTTAYGACTT |
| XLR_1492R_v2bBar1292L | AACCGTGTC | CCATCTCATCCCTGCGTGTCTCCGACTCAGAACCGTGTCTACGGYTACCTTGTTAYGACTT |
